# The kidney failure risk equation predicts kidney failure: Validation in an Australian cohort

**DOI:** 10.1111/nep.14160

**Published:** 2023-04-19

**Authors:** Georgina L. Irish, Laura Cuthbertson, Alex Kitsos, Tim Saunder, Philip A. Clayton, Matthew D. Jose

**Affiliations:** ^1^ Australia and New Zealand Dialysis and Transplant (ANZDATA) Registry South Australian Health and Medical Research Institute (SAHMRI) Adelaide Australia; ^2^ Central and Northern Adelaide Renal and Transplantation Service Royal Adelaide Hospital Adelaide Australia; ^3^ Department of Medicine The University of Adelaide Adelaide Australia; ^4^ School of Medicine University of Tasmania Australia; ^5^ Renal Unit, Royal Hobart Hospital Tasmanian Health Service Tasmania Australia

**Keywords:** Australia, calibration, chronic, kidney, renal dialysis, renal insufficiency

## Abstract

**Aims:**

Predicting progression to kidney failure for patients with chronic kidney disease is essential for patient and clinicians' management decisions, patient prognosis, and service planning. The Tangri et al Kidney Failure Risk Equation (KFRE) was developed to predict the outcome of kidney failure. The KFRE has not been independently validated in an Australian Cohort.

**Methods:**

Using data linkage of the Tasmanian Chronic Kidney Disease study (CKD.TASlink) and the Australia and New Zealand Dialysis and Transplant Registry (ANZDATA), we externally validated the KFRE. We validated the 4, 6, and 8‐variable KFRE at both 2 and 5 years. We assessed model fit (goodness of fit), discrimination (Harell's C statistic), and calibration (observed vs predicted survival).

**Results:**

There were 18 170 in the cohort with 12 861 participants with 2 years and 8182 with 5 years outcomes. Of these 2607 people died and 285 progressed to kidney replacement therapy. The KFRE has excellent discrimination with C statistics of 0.96–0.98 at 2 years and 0.95–0.96 at 5 years. The calibration was adequate with well‐performing Brier scores (0.004–0.01 at 2 years, 0.01–0.03 at 5 years) however the calibration curves, whilst adequate, indicate that predicted outcomes are systematically worse than observed.

**Conclusion:**

This external validation study demonstrates the KFRE performs well in an Australian population and can be used by clinicians and service planners for individualised risk prediction.

## Summary at a Glance

- The Kidney Failure Risk Equation (KFRE) predicts kidney failure, but has never been validated in Australia.
- The KFRE score has excellent discrimination and adequate calibration. We developed an Australian recalibration factor to allow better prediction.
- This study confirms that the KFRE performs well and can be used for decision‐making.

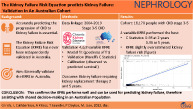

## 1 INTRODUCTION

Chronic Kidney Disease (CKD) is common with an increasing incidence globally.^1^ Patients with CKD, however, represent a highly heterogeneous cohort. There is great variation in rates of disease progression, with the prevalence of patients with CKD who reach kidney failure being 0.1% of all patients.^2^ Being able to predict who will progress to kidney failure is important at a patient level, a clinician level, and a policy level. Understanding the risk of progression to kidney failure, allows patients and clinicians to make informed decisions about future care: ranging from the implementation of measures to slow kidney function decline, decisions about kidney replacement therapy (KRT) including when to start dialysis education, the timing of arteriovenous fistula creation and pre‐emptive kidney transplantation.^3^, ^4^ It is also important on a population level for resource allocation and workforce planning. This need for prognostication has led to the need for more individualised prediction tools. The use of validated risk prediction tools is now recommended by the international Kidney Disease Improving Global Outcomes (KDIGO) guidelines.^5^

Tangri et al developed a kidney failure risk prediction tool or equation within a Canadian cohort of CKD patients in 2011.^4^ The Kidney Failure Risk Equation (KFRE) includes the use of routinely obtained demographic and pathology data to predict the progression of CKD to kidney failure (starting KRT). The KFRE was validated within the Canadian population and found to be more accurate at predicting progression to kidney failure than the previously used combination of estimated glomerular filtration rate (eGFR) and albuminuria alone.^4^ The utility of the KFRE prediction model was further validated in a multinational assessment of 31 cohorts spanning 4 continents, which demonstrated high discrimination and adequate calibration.^6^ This study determined that for some populations the equation needed a calibration adjustment factor before use.^6^ The KFRE, however, has not been independently validated in an Australian population.

There are differences between the Australian^7^ and Canadian cohorts with differences in clinical practice.^8^ It is therefore unclear whether we can apply the score in our population without a calibration adjustment factor. The KFRE is available online^9^ and its simplicity means it can be automated into electronic health records.^10^ Several potential uses are identified including triaging for speciality nephrology care, identification of people who are at high risk of CKD progression and treatment planning.^11^ The purpose of this study is to externally validate the KFRE in an Australian cohort, to evaluate its performance and therefore whether it can be used to guide management decisions for patients and clinicians in the Australian population.

## 2 METHODS

### 2.1 Study population

The Tasmanian Chronic Kidney Disease study (CKD.TASlink) is a retrospective cohort study of a dataset created through linkage of seven existing local health information datasets. Detailed methods and linked data obtained are available in separate methodology papers.^12^, ^13^ Linkage to the Australia and New Zealand Dialysis and Transplant Registry (ANZDATA), the Tasmanian public hospital admitted patient dataset, Tasmanian public hospital emergency presentation dataset, Tasmanian cancer registry and the Tasmanian death registry was performed by the Tasmanian Data Linkage Unit (see supplementary methods).

## 3 LINKAGE

Linkage of individual addresses to the Geocoded National Address File (G‐NAF), then allocated to the statistical area (SA2). Linkage to the Australian and New Zealand Dialysis and Transplant Registry were from 01/01/2004–31/12/2019. Diagnosis of CKD was thus between 01/01/2004–31/12/2019. Pathology data on individuals was obtained from community and hospital‐based pathology providers between 1/1/2004–31/12/2020 to allow 1 year of follow‐up. Inclusion criteria were any individual diagnosed with CKD, over the age of 18, and resident within Tasmania (based on SA2 geocode records). Hypertension was derived from International Classification of Disease (ICD)‐10 diagnosis codes via Elixhauser comorbidity categories (for patients who had hospital admission where this diagnosis was coded). Diabetes was derived from ICD‐10 diagnosis codes via Elixhauser comorbidity categories, or if a pathology result for Haemoglobin A1C > 6.5% or Fasting Glucose >7.0 mmol/L was recorded. The 6 variable equations rely on the past medical history of diabetes or hypertension. For this cohort we only used those who had linked data.

### 3.1 Diagnosis of CKD

Chronic kidney disease in individuals 18 years and older was defined using KDIGO criteria,^5^ requiring two eGFR <60 mL/min/1.73m^2^ 90 days apart. CKD severity was categorised by eGFR and urine ACR (uACR) by the KDIGO CKD staging. Tasmanian laboratories use enzymatic assays for measurement of creatinine, and immunoassay for urinary albumin, all results are isotope dilution mass‐spectrometry (IDMS) aligned as previously reported.^14^ eGFR was calculated using the 2009 CKD‐EPI creatinine equation.^15^ Those who had had a previous kidney transplant were excluded from the initial cohort.

### 3.2 Risk scores

The three Tangri et al KFRE were calculated, at the date of CKD diagnosis for those with adequate data. The KFRE predicts the outcome of progression to kidney failure, defined as the need for KRT and censored for death prior to kidney failure.

**KFRE 4‐variable model:** age, sex (male), eGFR and uACR.^4^

**KFRE 6‐variable model:** addition of Diabetes and Hypertension.

**KFRE 8‐variable model:** addition of calcium, bicarbonate, phosphate, and albumin.

### 3.3 External validation

We externally validated the KFRE equations at 2 and 5‐years using 3 measures^10^; (i) Model fit (ii) Discrimination and (iii) Calibration.

Model fit: Model fit is how well the statistical model fits the outcome. We visually assessed this using goodness of fit and plotting the predicted risk compared to the observed risk.

Discrimination: Discrimination is how well a model can differentiate between individuals for an outcome.^10^, ^16^ We assessed discrimination of the KFRE using the Harrell's C statistic.^17^ This concordance statistic is a measure of discrimination; a value of 0.5 indicates no difference to chance alone and 1 indicates perfect discrimination.

Calibration: Calibration is how accurately the score predicts an outcome. To allow comparison of risk groups the KFRE scores were categorised into risk quintiles for both 2‐year and 5‐year risk scores with levels based on the original Tangri et al paper.^4^ We assess calibration in two ways:

1. Plotting the observed 2‐year and 5‐year probability of KRT and comparing it to predicted risk derived from the risk equations.
2. Calculation of the Brier Scores, the mean squared difference between the predicted risk vs observed binary outcomes. A Brier score of 0 indicates perfect calibration and a score of 1 indicates no calibration.^18^

### 3.4 Recalibration

Equations used in different populations can require recalibration for different demographic and geographic setting. We recalibrated the equation, using a post‐estimation prediction of the survival function from the data.^19^

### 3.5 Sensitivity analysis: Alternate outcome events

Typically, the KFRE is used to predict the risk of proceeding to kidney failure requiring KRT. Due to the low utilisation of renal replacement therapy in Tasmania compared to other populations,^7^ two alternate endpoints (eGFR <10 and <7.5 mL/min/1.73m^2^) were implemented to ensure the robustness of results; the methods for these are described in the supplementary appendix. The cut‐off of 7.5 mL/min/1.73m^2^ was chosen based on the median eGFR start of 7.5 mL/min/1.73m^2^ for Australians starting Kidney Replacement Therapy^20^ and the cut‐off of 10 mL/min/1.73m^2^ aligns with the secondary outcome used when originally developing the equation.^6^

### 3.6 Sensitivity analysis: Missing data

For the cohort without a uACR result within the 12 month window of an eGFR, a uACR was imputed using the multiple imputation techniques with 5 imputations with R package *mice*.^21^ The arithmetic mean of the model risks produced was used to pool each person's imputations. The data were assumed to be missing at random.

### 3.7 Statistical analysis and ethics

The statistical analyses were performed using R statistical software.^22^ The C statistics were calculated using the pROC R package.^23^ The CKD.Taslink protocol was reviewed and approved by the Tasmanian Human Research Ethics Committee (Approved study H0016499).

## 4 RESULTS

**FIGURE 1:**
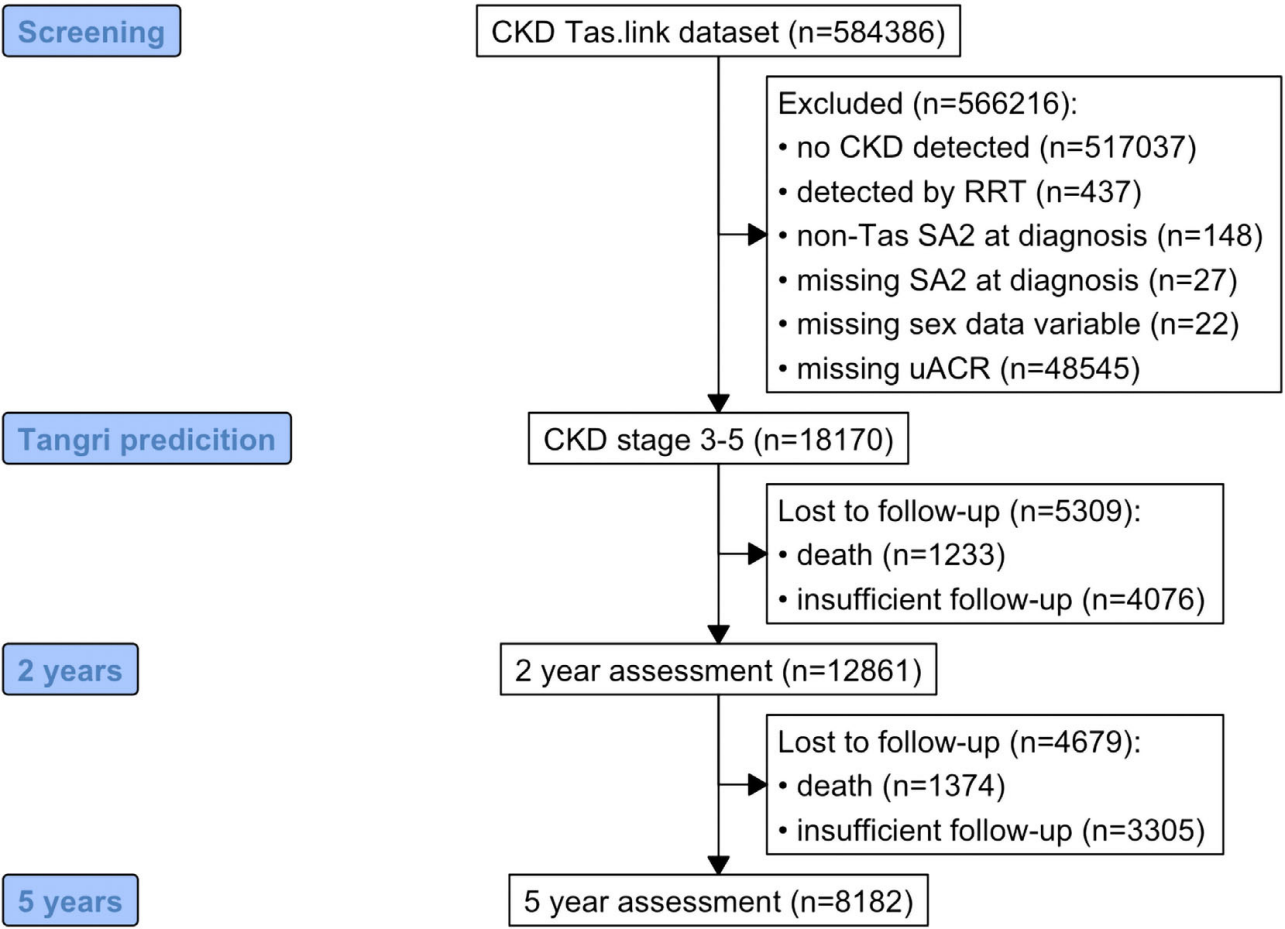
Consort diagram explaining study selection.

During the 16‐year study period, Figure 1 demonstrates 584 386 Tasmanian adults had a serum creatinine measured. Of these 18 170 had CKD stage 3–5, 12 861 had 2 years of follow up and 8182 had 5 years of follow‐up. Table 1 describes the demographics of the study population for the 4,6,8‐variable KFRE. The mean age at diagnosis of CKD was 70.7 (SD 10.4) years, there was a predominance of females (51.6%), 54.9% of people had diabetes and 41.2% had hypertension. The mean eGFR was 50.3 (SD 9.3) mL/min/1.73m^2^ and the median uACR was 1.4 [IQR 0.6–5] mg/g. Focusing on severity, at diagnosis only 159 (0.3%) had an uACR >300 mg/g and 100 (0.8%) had an eGFR of <15 mL/min/1.73m^2^. For outcomes, over the study period, 285 were treated with KRT (271 (2.1%) dialysis and 85 (0.7%) kidney transplantation) and 2607 people died. Table S1 demonstrates the proportion of people who were in each risk category for each KFRE and how many developed the outcome of KRT.

**TABLE 1:** Baseline characteristics for the cohort. SD standard deviation, eGFR estimated glomerular filtration rate, uACR urinary albumin: creatinine ratio, KRT kidney replacement therapy, IQR Interquartile range

Characteristics	4 ariable KFRE (*n* = 12 861)	6 variable KFRE (*n* = 10 429)	8 variable KFRE (*n* = 4969)
Age at diagnosis (years) ‐ mean (SD)	70.7 (10.4)	70.8 (10.6)	68.3 (11.9)
Sex (Female)	6635 (51.6%)	5314 (51.0%)	2479 (49.9%)
Diabetes	7064 (54.9%)	6146 (58.9%)	2635 (53.0%)
Hypertension	5297 (41.2%)	5297 (50.8%)	2414 (48.6%)
Pathology			
eGFR mean (SD) (mL/min/1.73m^2^)	50.3 (9.29)	49.9 (9.57)	48.3 (11.0)
eGFR categories (mL/min/1.73m^2^)			
<15	100 (0.8%)	93 (0.9%)	86 (1.7%)
15–29	469 (3.6%)	415 (4.0%)	299 (6.0%)
30–59	12 292 (95.6%)	9921 (95.1%)	4584 (92.3%)
uACR median [IQR] (mg/g)	1.40 [0.600–5.00]	1.60 [0.600–5.70]	1.90 [0.700–8.70]
uACR Categories (mg/g)			
<30	11 748 (91.3%)	9429 (90.4%)	4275 (86.0%)
30–299	950 (7.4%)	846 (8.1%)	560 (11.3%)
≥300	159 (1.2%)	152 (1.5%)	132 (2.7%)
Outcomes			
KRT	285 (2.2%)	276 (2.6%)	202 (4.1%)
Dialysis	271 (2.1%)	262 (2.5%)	191 (3.8%)
Transplant	85 (0.7%)	84 (0.8%)	69 (1.4%)

### 4.1 Goodness of fit

**FIGURE 2:**
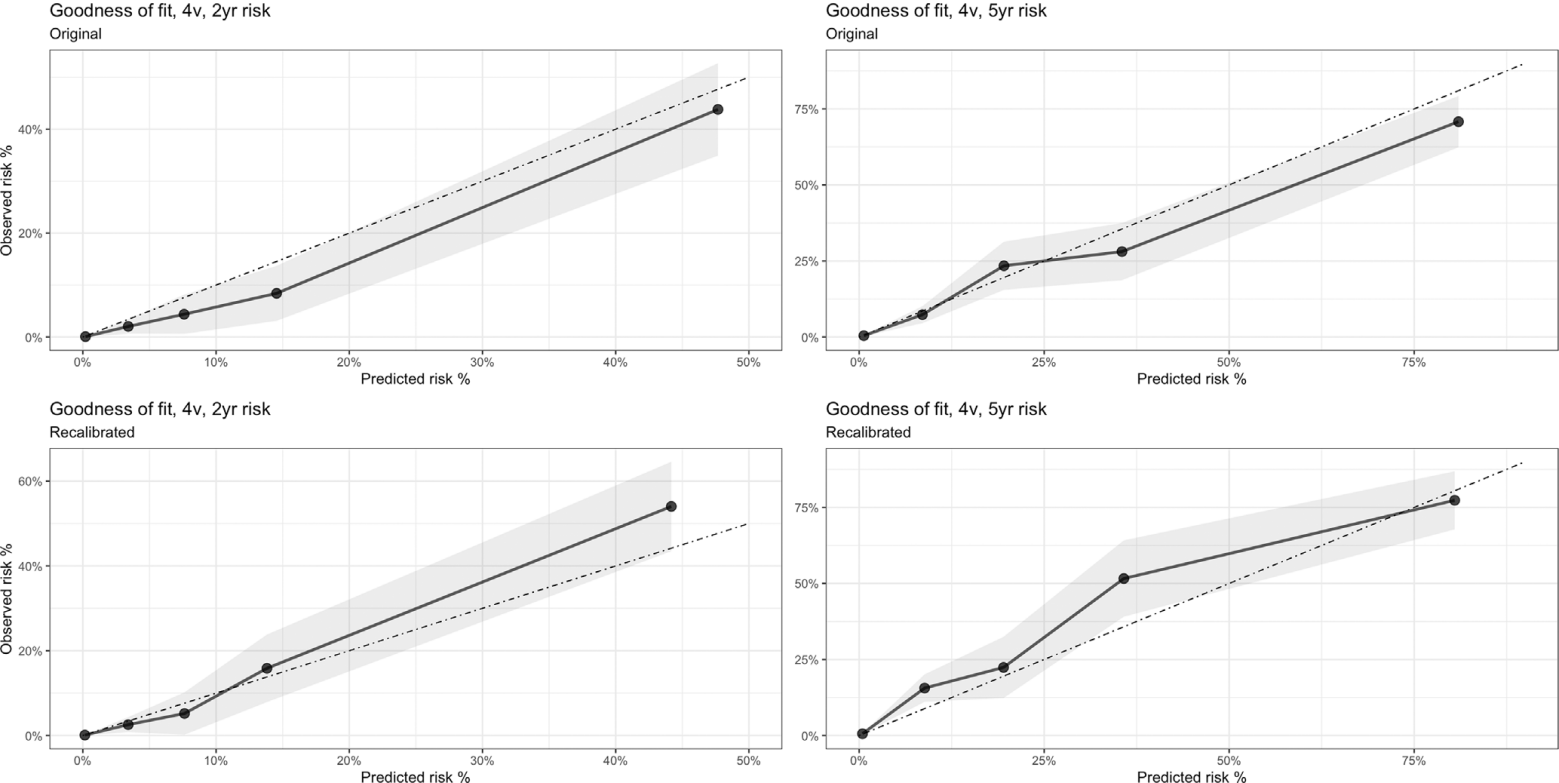
Original 4 variable model before and after re‐calibration demonstrating goodness of fit by risk group for the KFRE at 2 and 5 years.

Figure 2 and s1 demonstrates the goodness of fit for the observed compared to predicted risk of the scores showing some misspecification with the predicted risk being more than the observed at 2 and 5 years for all equations. A perfectly fit model will align with the 45‐degree line.

### 4.2 Discrimination

Table 2 demonstrates the C statistics for the different KFRE. All of the KFRE performed well with excellent discrimination with the C statistics at 2 years were 0.96–0.98 and 0.95–0.96 at 5 years.

**TABLE 2:** Demonstrates Harrell's C statistics for the 4, 6 or 8 variables KFRE at 2 and 5 years of follow‐up. (KFRE Kidney Failure Risk Equation, CI Confidence Interval)

C statistic [95% CI]		
KFRE Model	2 years	5 years
4 variables	0.98 [0.97–0.99]	0.96 [0.95–0.97]
6 variables	0.97 [0.96–0.99]	0.96 [0.95]4–0.97]
8 variables	0.96 [0.94–0.98]	0.95 [0.93–0.96]

### 4.3 Calibration

Calibration was assessed in two ways, visually and quantitatively using Brier scores. The calibration performed well on quantitively assessment but demonstrates some miscalibration on visual assessment.

**FIGURE 3:**
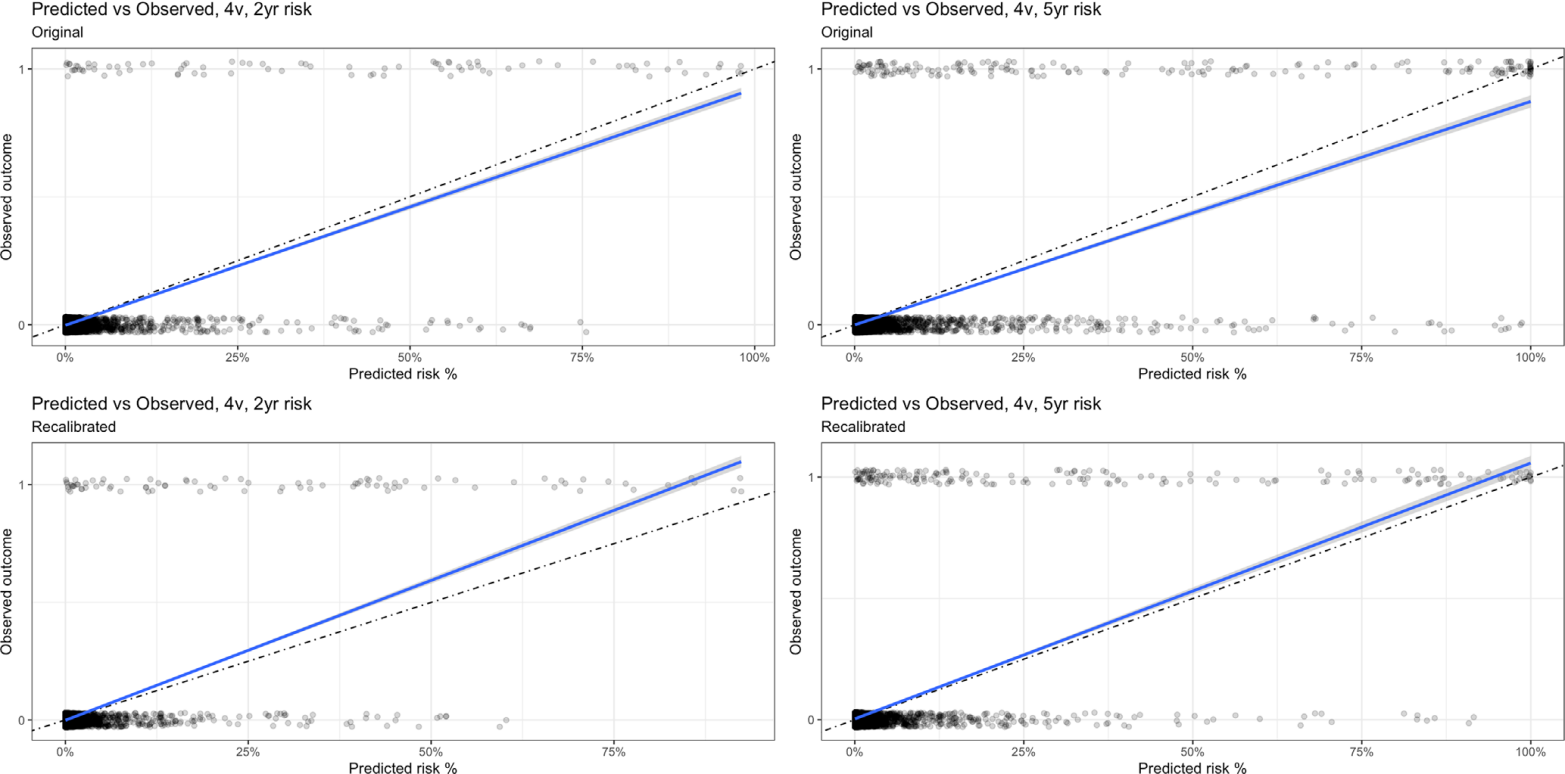
Original 4 variable model before and after re‐calibration demonstrating predicted risk vs observed outcomes by risk group for the KFRE at 2 and 5 years.

1. Visually, this is demonstrated with Figures 3 and s2‐3 of the observed risk vs the predicted outcomes graphed continuously and by risk groups. The visual assessment of calibrations demonstrate that all scores had a degree of miscalibration, but this was worse for the 8 variable KFRE for those with >50% of the risk of progression.
2. Quantitatively all the KFRE scores had good calibration. The Brier scores are demonstrated in Table 3 and show very low scores for all the KFRE (0 is perfect calibration and 1 is no calibration) with all scores being less than 0.02. All 2‐year scores had better calibration that the 5‐year scores, but this difference was not marked (2‐year 0.004–0.01 compared to 5‐year 0.01–0.03). The worst calibration was again from the 8‐variable KFRE at 5 years 0.03 however this is still considered good calibration.

**TABLE 3:** Brier scores were used to evaluate which risk equation showed the best calibration for each model. A Brier score of 0 is perfect calibration and 1 is no calibration. All models are well calibrated. (KFRE kidney failure risk equation)

	2 years		5 years	
KFRE Model	Number of patients	Brier score	Number of patients	Brier score
4 variables	12 861	0.004	8182	0.01
6 variables	10 429	0.005	6676	0.02
8 variables	4969	0.01	2877	0.03

### 4.4 Recalibration

The KFRE model may benefit from a recalibration whereby the baseline survival rate by updated to represent the local cohort. The adjusted baseline survival for recalibration was calculated through post‐estimation prediction of the survival function from the cohort's data.

Table 4 demonstrates the baseline survival function for the original and recalibrated model which can be used for recalibration. Figure 2, 3 demonstrates the improvement with recalibration.

**TABLE 4:** Demonstrates the baseline survival for the original model compared to the recalibrated model. This can be used to recalibrate the KFRE for a Tasmanian cohort

Baseline survival		
Risk period	Original model	Recalibrated model
2 Year	0.9832	0.9890
5 Year	0.9365	0.9633

### 4.5 Sensitivity analysis: Alternative outcomes

The sensitivity analysis demonstrated similar outcomes.

Model fit: Figure s4 demonstrated the model fit for the different outcomes. The outcome of KRT or eGFR <7.5 mL/min/1.73m^2^ had the best fit with the observed risk vs predicted risk overlying the 45‐degree angle.

Discrimination: Table s2 demonstrates the C statistics for the alternative endpoints of KRT or eGFR <10 mL/min/1.73m^2^ and KRT or eGFR <7.5 mL/min/1.73m^2^. The discrimination is lower with the alternative endpoints 0.87–0.92 compared to 0.96–0.98 however would still be considered strong discrimination confirming the robustness of this finding.

Calibration: Figure s5 demonstrates the calibration for observed risk compared to predicted outcomes for the KFRE scores. For 2 years of follow‐up, the 4 and 6‐variable KFRE performed better than the 8‐variable model. For the 5‐year risk prediction, all models were somewhat miscalibrated with predicted outcomes being worse than observed, though the 8‐variable risk score had poorer calibration. Figures s6‐s8 demonstrate the observed vs predicted probabilities for the different risk scores. Again the 8‐variable models performed worse than the 4 and 6‐variable models. Table s3 tabulates the Brier scores for all the different models with alternative outcomes. This shows that the Brier score were best with the original outcome of KRT but were still low for all alternative outcomes less than or equal to 0.05 indicating the scores were well calibrated.

### 4.6 Sensitivity analysis: Missing data

For a sensitivity analysis we undertook multiple imputations for the missing uACR data 33 976 (72%).

for the 4 variable KFRE and 11 012 (68.9%) for the 8‐variable KFRE. The outcomes were similar for model fit, discrimination and calibration using multiple imputation. Detailed results of the sensitivity analysis can be found in the supplementary appendix in Table s4‐s5, and Figures s9‐s11.

## 5 DISCUSSION

When validated within an Australian population, the KFRE has excellent discrimination with C statistics of 0.95–0.98, is slightly mis‐specified for the goodness of fit and is well calibrated with Brier scores from 0.004–0.03. This suggests that the KFRE can be used within an Australian context to predict the outcome of the need for KRT. The 4 and 6‐variable KFRE performed better than the 8‐variable risk score. This, plus the simplicity of the 4 and 6‐variable scores, suggest these should be preferentially used for risk prediction.

The KFRE has been validated in many countries but not previously in an independent Australian cohort.^6^, ^19^, ^24^, ^25^, ^26^, ^27^, ^28^ The results of excellent discrimination and adequate calibration align with the original publication.^4^ The original C statistics were similar at 0.90 (95% CI 0.89–0.92) and 0.8836 (0.87–0.90) for the 4‐variable equation at 2 and 5 years. Similarly, a large meta‐analysis of 31 cohorts demonstrated similar results for discrimination with pooled C statistic of 0.90 (95%CI 0.89–0.92) at 2 years and slightly worse discrimination at 5 years with and 0.88 (95% CI 0.86–0.90) for the 4‐variable equations. A number of other studies from Canada, the United Kingdom, Singapore, Korea and Europe have also validated the KFRE and they all showed similarly strong discrimination with C‐statistics from 0.83–0.93.^19^, ^25^, ^26^, ^27^, ^28^

For calibration, the KFRE overestimates risk in our population. The Brier score in our cohort was better than in the multinational validation study for non‐north American results with 0.228–0.299. This may be due to the non‐north American cohort being a more heterogenous population as it was made up cohorts from multiple different countries.^6^ Recalibration, with use of a calibration factor to adjust for systematic differences, can account for the differences in clinical practice. Interestingly, all the independent studies that validated the KFRE showed some degree of miscalibration with overprediction of risk.^19^, ^25^, ^26^, ^27^, ^28^ Three of these studies^18,23,24^ and the large validation study by Tangri et al^6,^ undertook recalibration for use in an external population.^19^, ^25^, ^26^ Previous work has demonstrated differences in practice between Australia and Canada in regards to eGFR and commencement of dialysis.^7^, ^8^ To account for this we have analysed several alternative endpoints which have demonstrated similar findings for discrimination to demonstrate the robustness of our results. For calibration, this has demonstrated that the observed and predicted risk are better aligned when using the outcome of <7.5 mL/min/1.73m^2^ which further highlight the differences in dialysis initiation practices between systems.

Risk prediction scores have several key purposes; informing patients about expected disease course and prognosis, informing individual clinical healthcare decisions, stratification of risk for clinical trials, and assessing healthcare systems.^10^ From a patient's perspective, risk scores can improve risk perception and increase intent for behavioural change to reduce risk.^29^ Our study has demonstrated that few people progress to requiring KRT, and thus this score may assist with alleviating patient anxiety. However, further work exploring consumers' understanding of the risk calculator and whether this could form part of a patient decision aid to improve understanding needs to be explored.^30^ The clinical impact of incorporating the KFRE into routine care is still being studied^11^ and further work is needed to assess the impact of the score on patient‐important outcomes and the cost‐effectiveness of implementation.

This study has several strengths. It is a large validation study that has not previously been performed within an Australian population which may have system‐level differences from the original Tangri et al cohort. The estimated Tasmanian resident population (ERP) in 2020 was 540 780 of which 409 729 were aged 18 years and older.^31^ Previous work showed the individuals identified within the CKD.Taslink dataset equated to 47.6%, 74.2% or 86.8% of the adult Tasmanian population overall when considered over 1, 3 or 5 years respectively in the years 2013–17– other unpublished work by this group suggests these proportions are similar.^12^ As this is a pathology dataset based on serum creatinine, annual representation varies by age, with 82.5% of Tasmanian 80 to 84‐year‐olds represented in 2017, but only 7.8% of people under 18 years.^12^ This older age group represented in the study represents the age group with the greatest prevalence of CKD. The use of data linkage of routine pathology results supports that the KFRE can be applied in primary care as well as in nephrology practice. This is particularly important as a great proportion of those with CKD are unlikely to see specialistic nephrology services.^32^ We have undertaken an extensive validation study of 4,6, and 8 KFRE with multiple sensitivity analyses to ensure the robustness of our findings.

There are some weaknesses to the study. Despite the increased prevalence of CKD, Tasmania has the lowest incidence of kidney failure treated with dialysis or transplantation of any Australian state or territory.^20^ In 2018 the incident rate of KRT was 87 per million population (pmp) in Tasmania, but 124pmp for Australia overall.^12^ This may be due to practice level differences with older Tasmanian being less likely to have KRT compared to other parts of Australia and may limit the generalisability of these data to the entire Australian population. The limitations of using hospital admissions data to define comorbidities may mean missing comorbidity data, however we have restricted the 6 variable equation to only use those with linked hospital data to try and address this. We used the sensitivity analysis of multiple imputation to deal with the missingness of the uACR data which is an accepted method to account for missing data and increase model power with no change in the outcomes.^33^

In conclusion, in this study, we have externally validated the KFRE and demonstrated exemplary discrimination and adequate calibration. This calibration further improved with recalibration adjustment factor. These findings support the use of the KFRE for risk prediction in an Australian population by patients and clinicians for clinical decision‐making as well as health service and workforce planning. Further work is needed on incorporating the KFRE into clinical care, and the resultant patient and health system outcomes.

## CONFLICT OF INTEREST STATEMENT

The authors of this manuscript have no conflicts of interest to disclose.

## Supporting information


**Data S1:** Supporting Information

## Data Availability

Restrictions apply to the availability of these data, which were used under licence for this study.
