# Supplementary material for: The kidney failure risk equation predicts kidney failure: Validation in an Australian cohort
Source: Nephrology (Carlton). 2023 Apr 19;28(6):328–35. doi: 10.1111/nep.14160 (PMC10946457; doi:10.1111/nep.14160)
Supplement: Supplementary file 1 — Data S1: Supporting Information [file NEP-28-328-s001.docx]

Supplementary Appendix

**Contents**

**Supplementary Tables**

Table s1: Demonstrates the proportion of people who were in each risk category all KFRE equations with 2 and 5 year outcomes and how many developed the outcome of KRT.

Table s2: Sensitivity analysis discrimination indicated by the C-statistics for the alternative outcomes. The C statistics are lower with the alternative outcomes at 2 and 5 years but still considered strong discrimination.

Table s3: Sensitivity analysis demonstrating the Brier scores for the alternative outcomes. Brier scores were used to evaluate which risk equation showed the best calibration for each model. A Brier score of 0 is perfect calibration and 1 is no calibration. All models are well calibrated. (n Number)

Table s4: Baseline demographics of cohort with multiple imputation

Table s5: Demonstrates the proportion of people who were in each risk category is use multiple imputation for uACR for all KFRE equations with 2 and 5 year outcomes and how many developed the outcome of KRT.

**Supplementary Figures**

Figure s1: Goodness of fit for the 4,6 and 8-variable KFRE at 2 and 5 years. The models are well fit, though less so for the 8 variables equations especially at the higher risk groups.

Figure s2: Calibration for the 4,6, and 8-variable KFRE at 2 and 5 years. The predicted risk is worse than the observed risk for all variables and time points, however is most miscalibrated for the 8-variable KFRE.

Figure s3: Calibration by risk group for the 4,6, and 8-variable KFRE at 2 and 5 years. The predicted risk is worse than the observed risk for all variables and time points, however is most miscalibrated for the 8-variable KFRE.

Figure s4: Sensitivity analysis for goodness of fit from different outcomes. This demonstrates the eGFR < 7.5 mL/min/1.73m^2^ has the best model fit with the line being closest to the 45 degree angle.

Figure s5: Sensitivity Analysis for different outcomes demonstrates calibration for the different KFRE models at 2 and 5 years. The 4 and 6 variable risk score were better calibrated than the 8 variable risk score.

Figure s6: Sensitivity Analysis for different outcomes demonstrates calibration for 4 variable KFRE models at 2 and 5 years by risk group.

Figure s7: Sensitivity Analysis for different outcomes demonstrates calibration for 6 variable KFRE models at 2 and 5 years by risk group.

Figure s8: Sensitivity Analysis for different outcomes demonstrates calibration for 8 variable KFRE models at 2 and 5 years by risk group.

Figure s9: Goodness of fit for the 4,6 and 8-variable KFRE at 2 and 5 years with multiple imputation .

Figure s10: Calibration for the 4,6, and 8-variable KFRE at 2 and 5 years with multiple imputation. The predicted risk is worse than the observed risk for all variables and time points, however is most miscalibrated for the 8-variable KFRE.

Figure s11: Calibration by risk group for the 4,6, and 8-variable KFRE at 2 and 5 years with multiple imputation . The predicted risk is worse than the observed risk for all variables and time points, however is most miscalibrated for the 8-variable KFRE.

| 2 years | | | | | | | | | |
| --- | --- | --- | --- | --- | --- | --- | --- | --- | --- |
|  | **4 variables** | |  | **6 variables** | |  |  | **8 variables** | |
| Risk category | No KRT | KRT | Total | No KRT | KRT | Total | No KRT | KRT | Total |
| <2% | 12117 | 10 | 12127 | 9764 | 10 | 9774 | 4356 | 7 | 4363 |
| 2% to <6% | 384 | 8 | 392 | 336 | 7 | 343 | 278 | 7 | 285 |
| 6% to <10% | 109 | 5 | 114 | 94 | 5 | 99 | 83 | 2 | 85 |
| 10% to <20% | 98 | 9 | 107 | 90 | 9 | 99 | 75 | 7 | 82 |
| ≥20% | 68 | 53 | 121 | 65 | 49 | 114 | 99 | 55 | 154 |
| Total | 12776 | 85 | 12861 | 10349 | 80 | 10429 | 4891 | 78 | 4969 |
| 5 years | | | | | | | | | |
|  | **4 variables** | |  | **6 variables** | |  |  | **8 variables** | |
| Risk category | No KRT | KRT | Total | No KRT | KRT | Total | No KRT | KRT | Total |
| <5% | 7495 | 34 | 7529 | 6053 | 35 | 6088 | 2354 | 14 | 2368 |
| 5% to <15% | 315 | 25 | 340 | 284 | 26 | 310 | 204 | 26 | 230 |
| 15% to <25% | 85 | 26 | 111 | 66 | 21 | 87 | 58 | 14 | 72 |
| 25% to <50% | 64 | 25 | 89 | 61 | 24 | 85 | 53 | 18 | 71 |
| ≥50% | 33 | 80 | 113 | 30 | 76 | 106 | 51 | 85 | 136 |
| Total | 7992 | 190 | 8182 | 6494 | 182 | 6676 | 2720 | 157 | 2877 |

*Table s1: Demonstrates the proportion of people who were in each risk category all KFRE equations with 2 and 5 year outcomes and how many developed the outcome of KRT.*

|  | C statistic for KFRE models [95% CI] | | | | | |
| --- | --- | --- | --- | --- | --- | --- |
|  | **KRT** | | **KRT or eGFR <10 mL/min/1.73m^2^** | | **KRT or eGFR <7.5 mL/min/1.73m^2^** | |
| KFRE | **2 years** | **5 years** | **2 years** | **5 years** | **2 years** | **5 years** |
| 4 variable | 0.98  [0.97-0.99] | 0.96  [0.95-0.97] | 0.88  [0.85-0.92] | 0.8  [0.84-0.90] | 0.92  [0.89-0.95] | 0.90  [0.88-0.93] |
| 6 variable | 0.97  [0.96-0.99] | 0.96  [0.94-0.97] | 0.88  [0.84-0.91] | 0.87  [0.86-0.89] | 0.91  [0.88-0.94] | 0.89  [0.87-0.92] |
| 8 variable | 0.96  [0.94-0.98] | 0.95  [0.93-0.96] | 0.90  [0.87-0.92] | 0.89  [0.86-0.91] | 0.91  [0.88-0.95] | 0.91  [0.88-0.93] |

*Table s2: Sensitivity analysis discrimination indicated by the C-statistics for the alternative outcomes. The C statistics are lower with the alternative outcomes at 2 and 5 years but still considered strong discrimination.*

|  | KRT | | | | | KRT or eGFR <10 mL/min/1.73m^2^ | | | | |  | | KRT or eGFR <7.5 mL/min/1.73m^2^ | | | |
| --- | --- | --- | --- | --- | --- | --- | --- | --- | --- | --- | --- | --- | --- | --- | --- | --- |
|  | | **2 years** | | **5 years** | | | **2 years** | | **5 years** | | | **2 years** | | | **5 years** | |
| Model | | **n** | **Brier** | **n** | **Brier** | | **n** | **Brier** | **n** | **Brier** | | **n** | | **Brier** | **n** | **Brier** |
| 4v | | 12861 | 0.004 | 8182 | 0.014 | | 12864 | 0.010 | 8225 | 0.027 | | 12853 | | 0.0073 | 8184 | 0.02 |
| 6v | | 10429 | 0.005 | 6676 | 0.017 | | 10431 | 0.012 | 6714 | 0.031 | | 10421 | | 0.009 | 6676 | 0.024 |
| 8v | | 4969 | 0.013 | 2877 | 0.035 | | 4973 | 0.021 | 2909 | 0.052 | | 4963 | | 0.0161 | 2878 | 0.042 |

*Table s3: Sensitivity analysis demonstrating the Brier scores for the alternative outcomes. Brier scores were used to evaluate which risk equation showed the best calibration for each model. A Brier score of 0 is perfect calibration and 1 is no calibration. All models are well calibrated however, the primary end point of KRT was better calibrated than the alternative outcomes. (n Number)*

| Characteristics | 4 Variable KFRE (n=46837) | 8 variable KFRE (n=15981) |
| --- | --- | --- |
| Age at diagnosis (years) - mean (SD) | 73.4 (10.5) | 72.0 (11.7) |
| Sex (Female) | 25669 (54.8%) | 8529 (53.4%) |
| Diabetes | 13704 (29.3%) | 4991 (31.2%) |
| Hypertension | 17011 (36.3%) | 6924 (43.3%) |
| Pathology |  |  |
| eGFR mean (SD) (mL/min/1.73^2^) | 49.8 (9.30) | 48.9 (10.4) |
| eGFR categories (mL/min/1.73^2^) |  |  |
| <15 | 306 (0.7%) | 227 (1.4%) |
| 15-29 | 1857 (4.0%) | 820 (5.1%) |
| 30-59 | 44674 (95.4%) | 14934 (93.4%) |
| uACR median [IQR] (mg/g) | 1.40 [0.60 - 5.00] | 1.90 [0.70 - 8.70] |
| uACR Categories (mg/g) |  |  |
| <30 | 11747 (25.1%) | 4275 (26.8%) |
| 30-299 | 950 (2.0%) | 560 (3.5%) |
| ≥300 | 159 (0.3%) | 132 (0.8%) |
| Outcomes |  |  |
| KRT | 762 (1.6%) | 391 (2.4%) |
| Dialysis | 714 (1.5%) | 366 (2.3%) |
| Transplant | 224 (0.5%) | 121 (0.8%) |

*Table s4:* *Baseline demographics of cohort with multiple imputation*

| 2 years | | | | | | | | | |
| --- | --- | --- | --- | --- | --- | --- | --- | --- | --- |
|  | **4 variables** | |  | **6 variables** | |  |  | **8 variables** | |
| Risk category | No KRT | KRT | Total | No KRT | KRT | Total | No KRT | KRT | Total |
| <2% | 44355 | 23 | 44378 | 44390 | 23 | 44413 | 14535 | 14 | 14549 |
| 2% to <6% | 1416 | 12 | 1428 | 1378 | 12 | 1390 | 688 | 9 | 697 |
| 6% to <10% | 345 | 15 | 360 | 353 | 16 | 369 | 193 | 2 | 195 |
| 10% to <20% | 320 | 20 | 340 | 316 | 21 | 337 | 175 | 20 | 195 |
| ≥20% | 206 | 125 | 331 | 205 | 123 | 328 | 224 | 121 | 345 |
| Total | 46642 | 195 | 46837 | 46642 | 195 | 46837 | 15815 | 166 | 15981 |
| 5 years | | | | | | | | | |
|  | **4 variables** | |  | **6 variables** | |  |  | **8 variables** | |
| Risk category | No KRT | KRT | Total | No KRT | KRT | Total | No KRT | KRT | Total |
| <5% | 28636 | 85 | 28721 | 28686 | 87 | 28773 | 8098 | 24 | 8122 |
| 5% to <15% | 1149 | 55 | 1204 | 1119 | 58 | 1177 | 549 | 39 | 588 |
| 15% to <25% | 319 | 48 | 367 | 304 | 41 | 345 | 138 | 20 | 158 |
| 25% to <50% | 234 | 81 | 315 | 232 | 86 | 318 | 134 | 31 | 165 |
| ≥50% | 98 | 180 | 278 | 95 | 177 | 272 | 126 | 178 | 304 |
| Total | 30436 | 449 | 30885 | 30436 | 449 | 30885 | 9045 | 292 | 9337 |

*Table s5: Demonstrates the proportion of people who were in each risk category is use multiple imputation for uACR for all KFRE equations with 2 and 5 year outcomes and how many developed the outcome of KRT.*

**
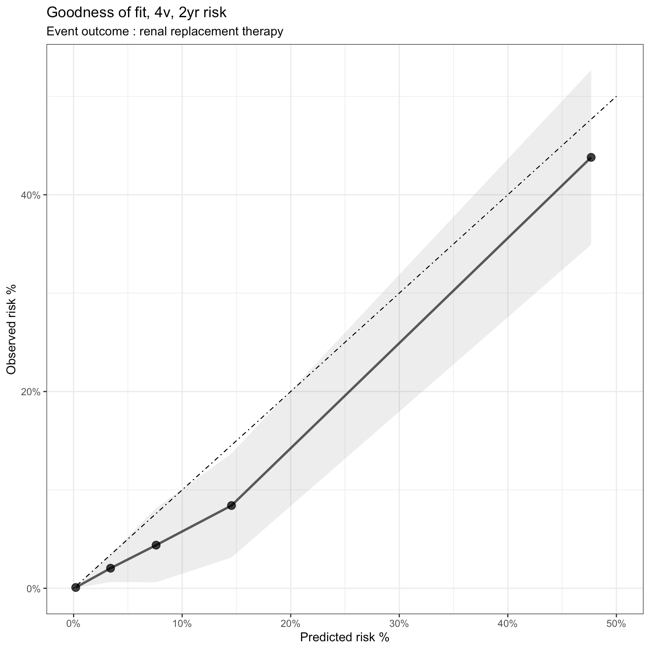

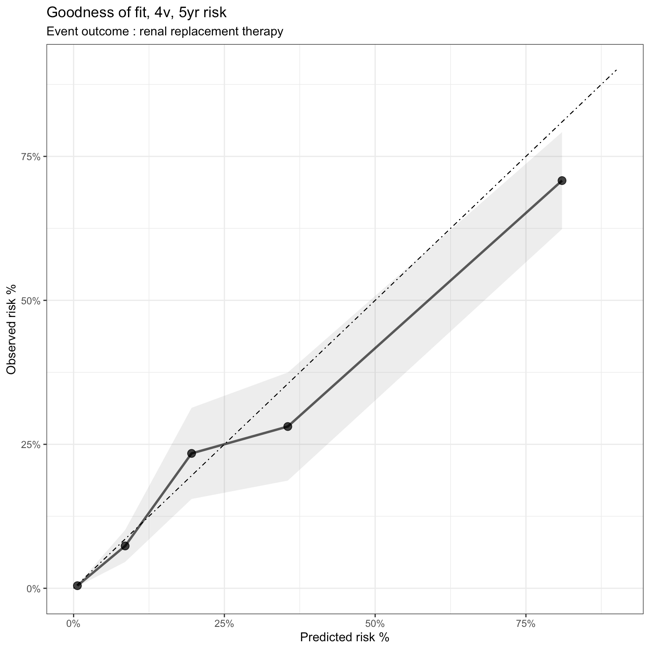
**

**
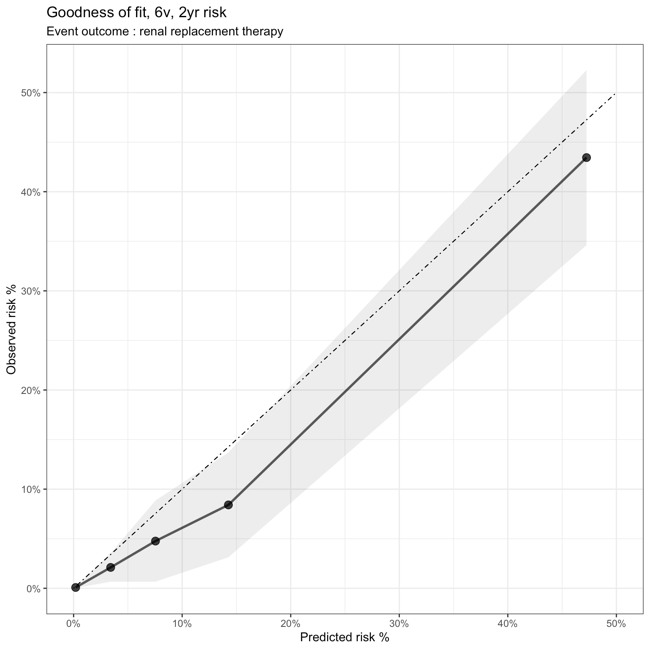

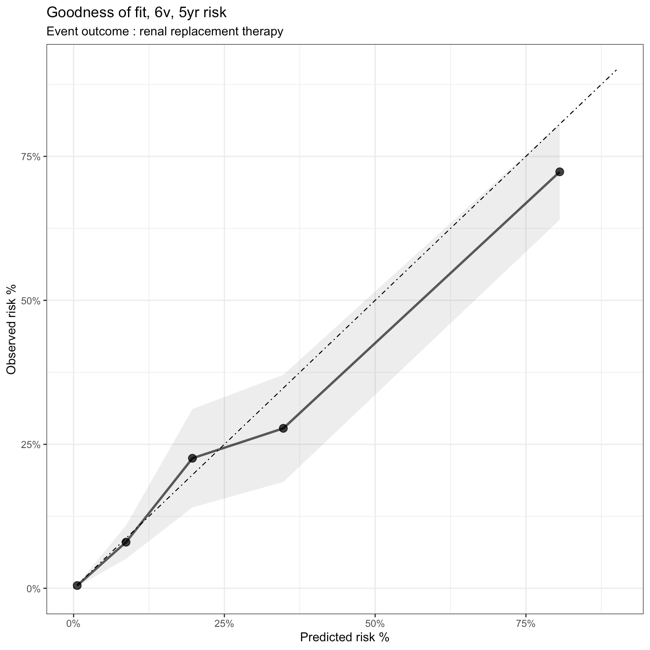
**

**
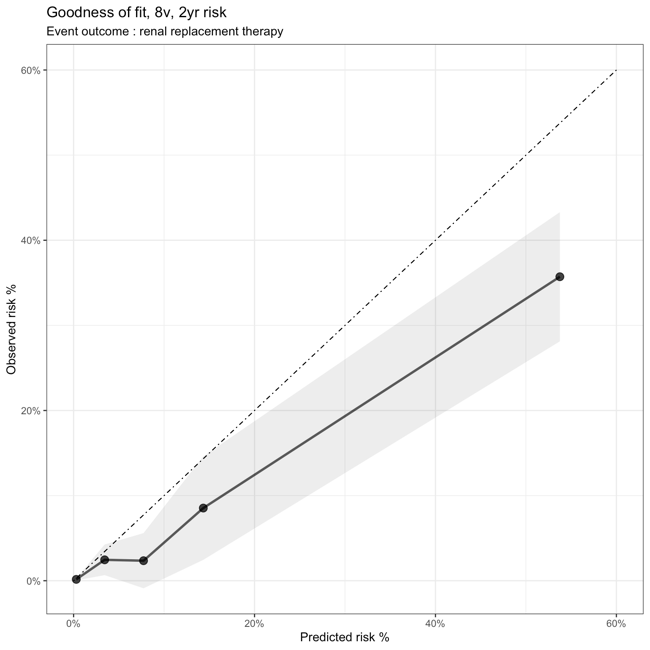

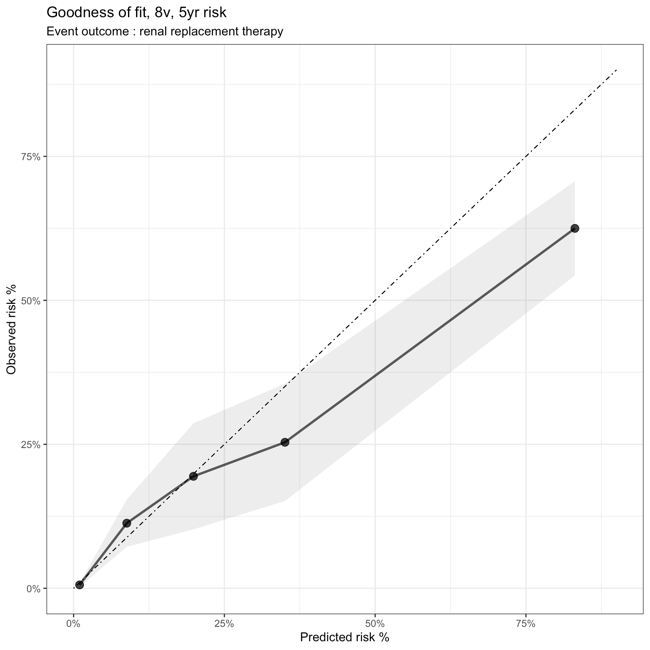
**

*Figure s1:Goodness of fit for the 4,6 and 8-variable KFRE at 2 and 5 years. The models are well fit, though less so for the 8 variables equations especially at the higher risk groups.*

*
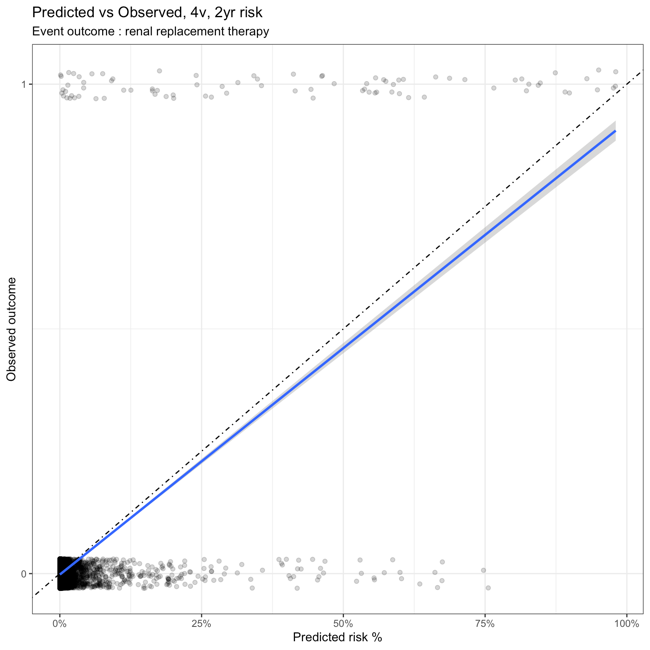
*
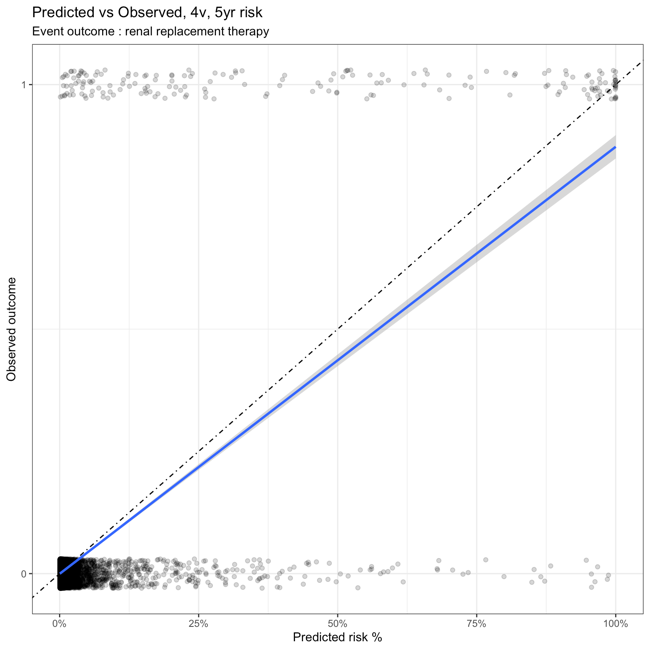


*
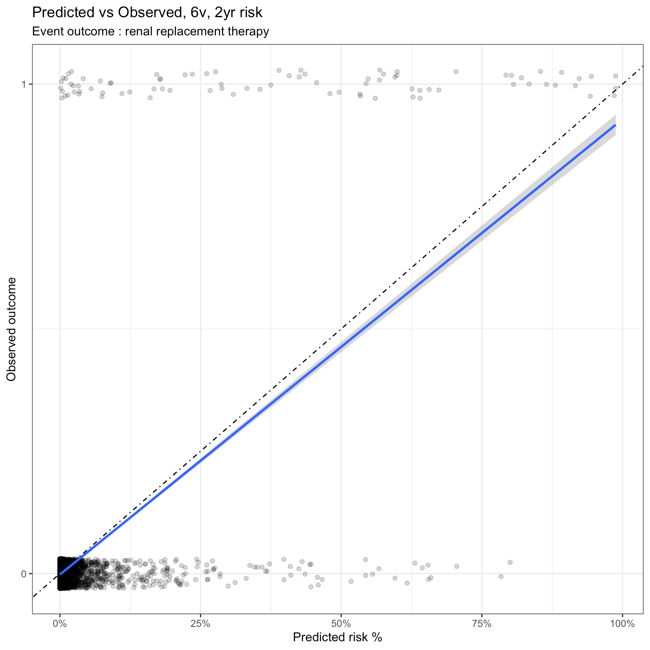

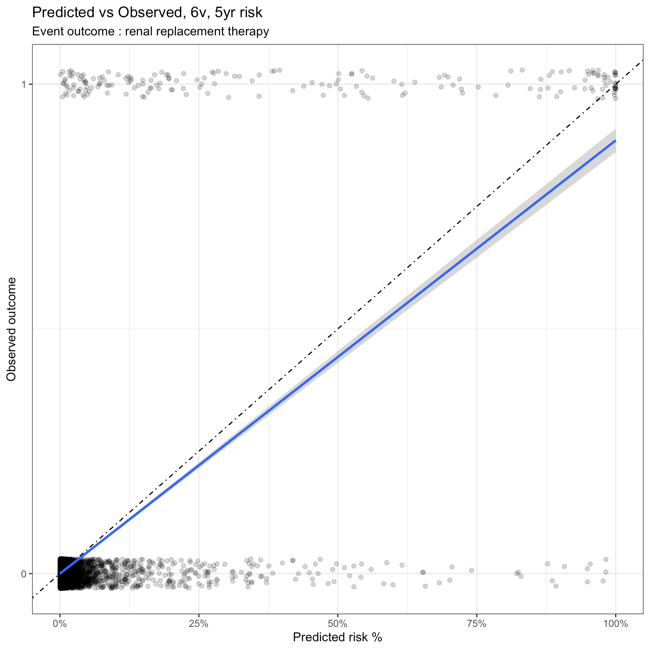
*

*
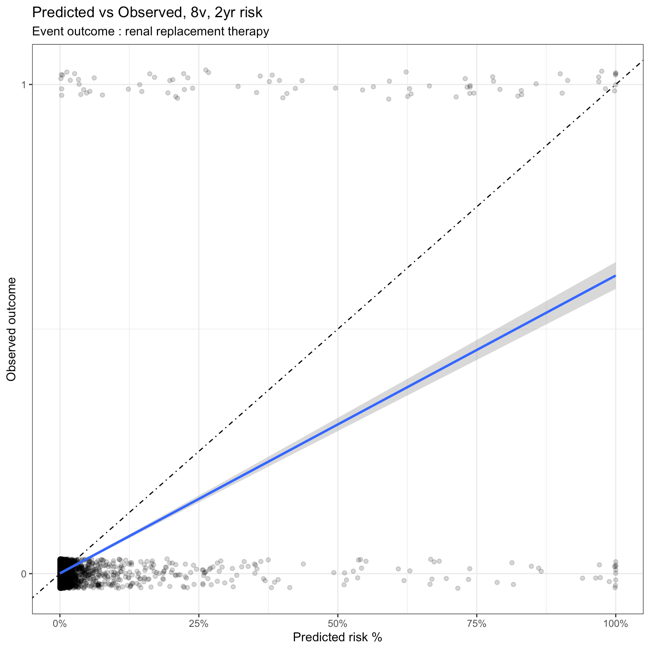

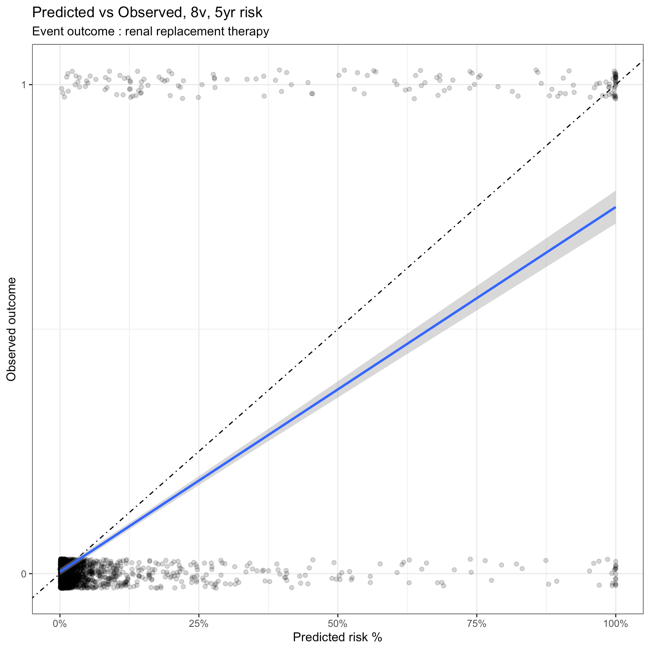
*

*Figure s2: Calibration for the 4,6, and 8-variable KFRE at 2 and 5 years. The predicted risk is worse than the observed risk for all variables and time points, however is most miscalibrated for the 8-variable KFRE.*


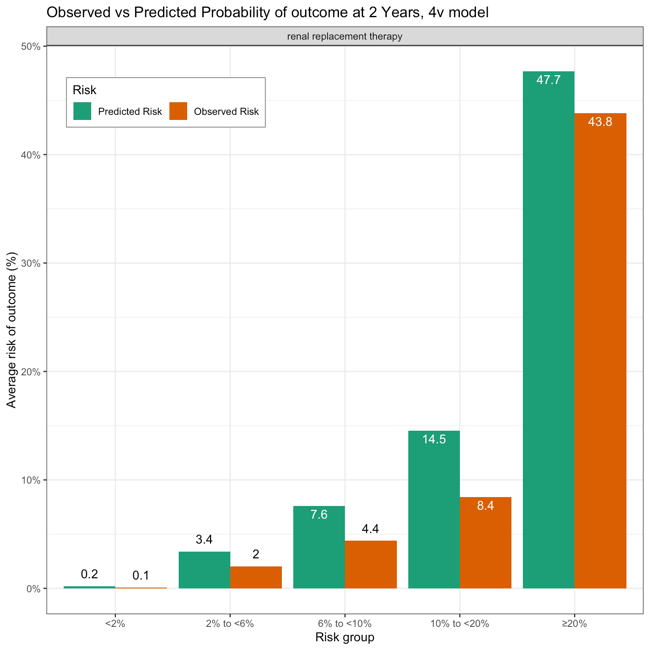

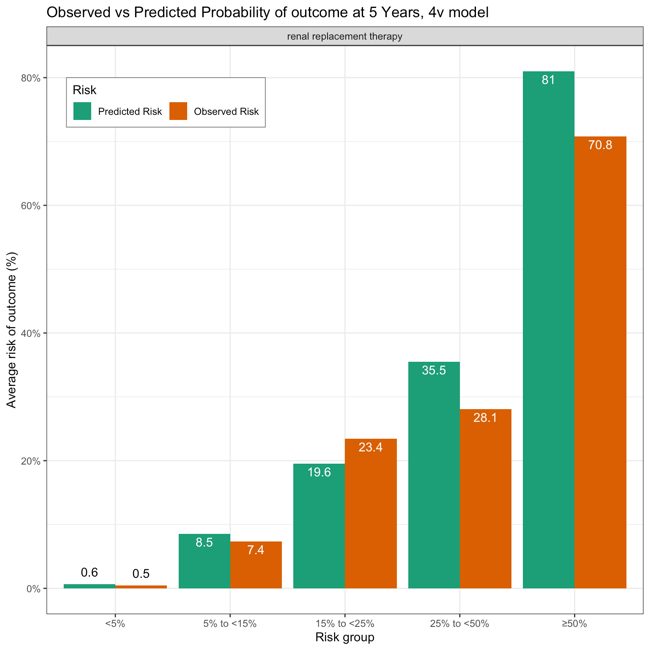


*
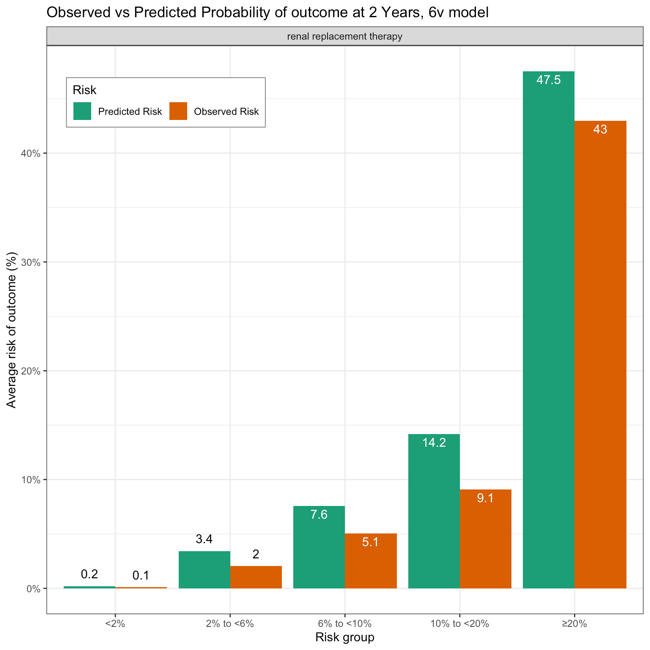

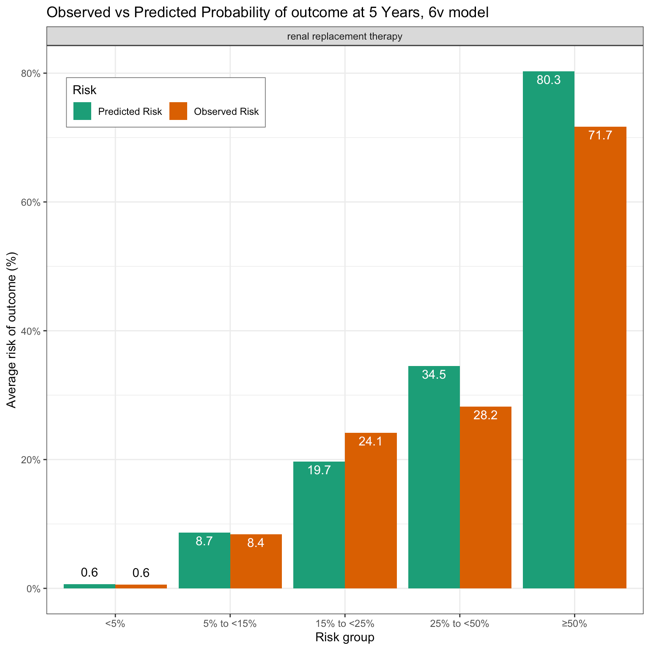
*

*
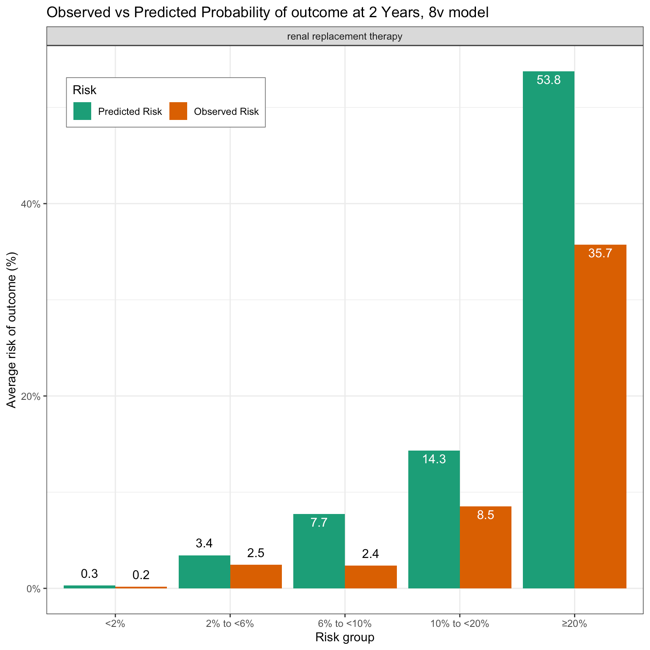

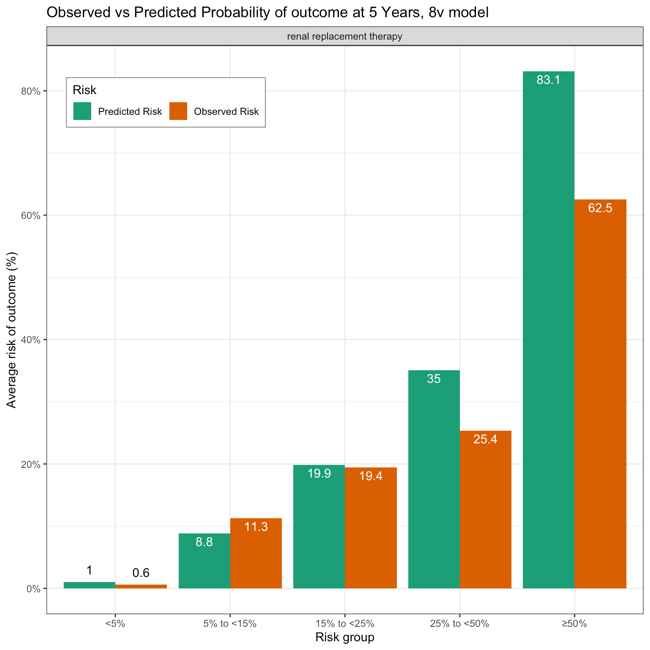
*

*Figure s3: Calibration by risk group for the 4,6, and 8-variable KFRE at 2 and 5 years. The predicted risk is worse than the observed risk for all variables and time points, however is most miscalibrated for the 8-variable KFRE.*

*
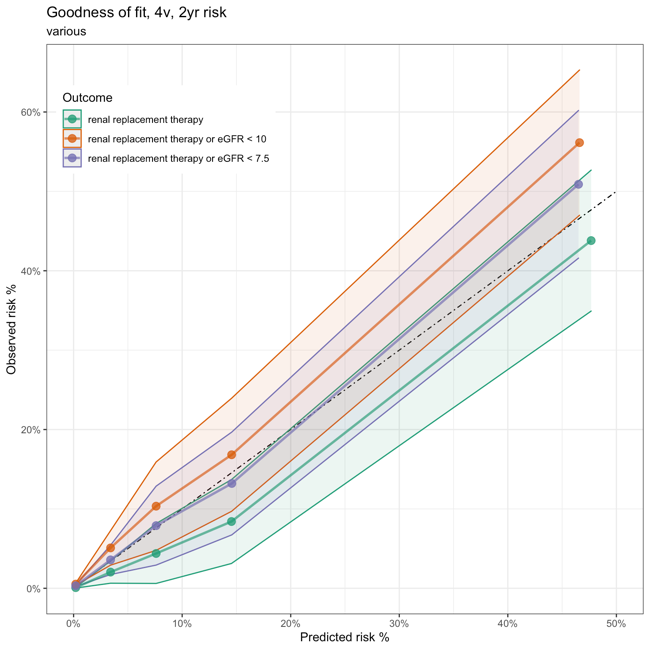

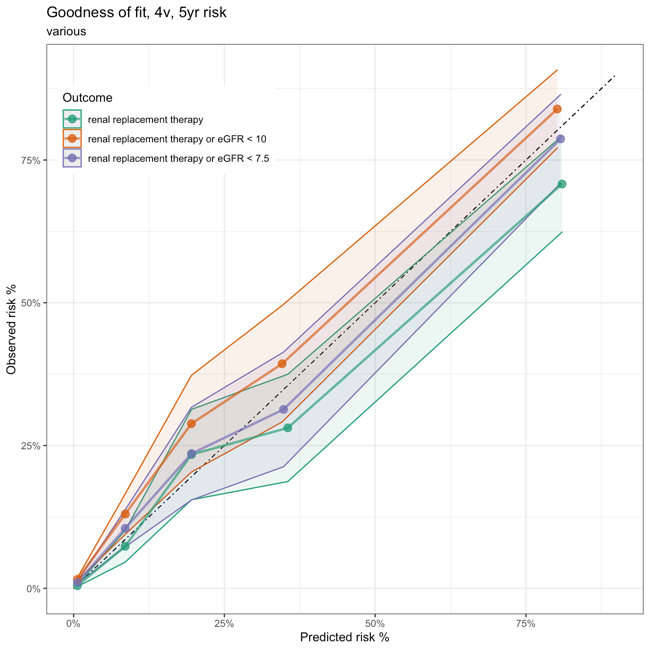
*

*
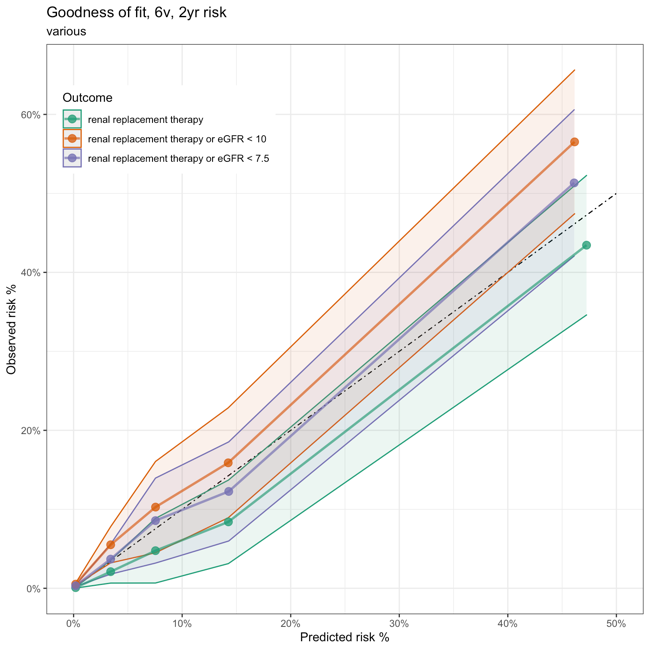

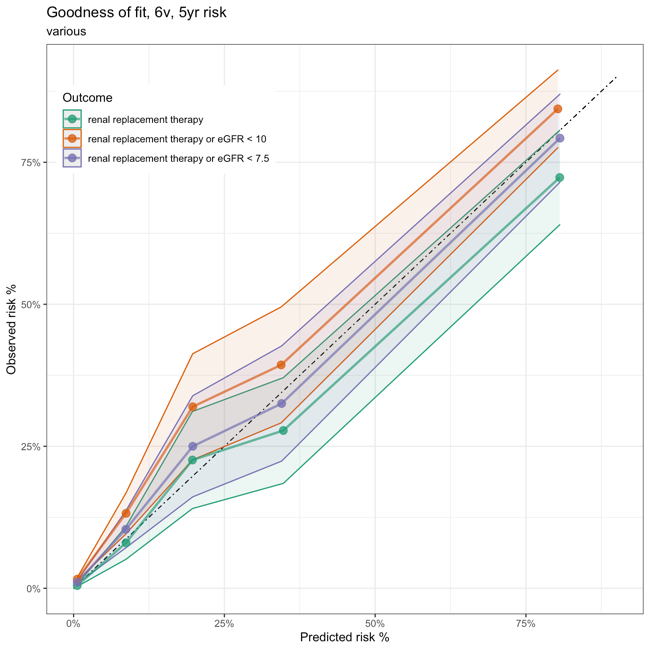
*

*
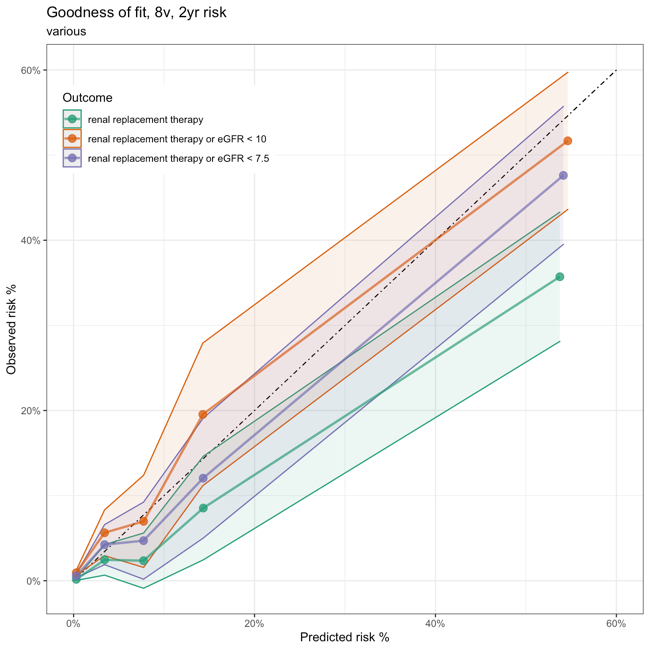

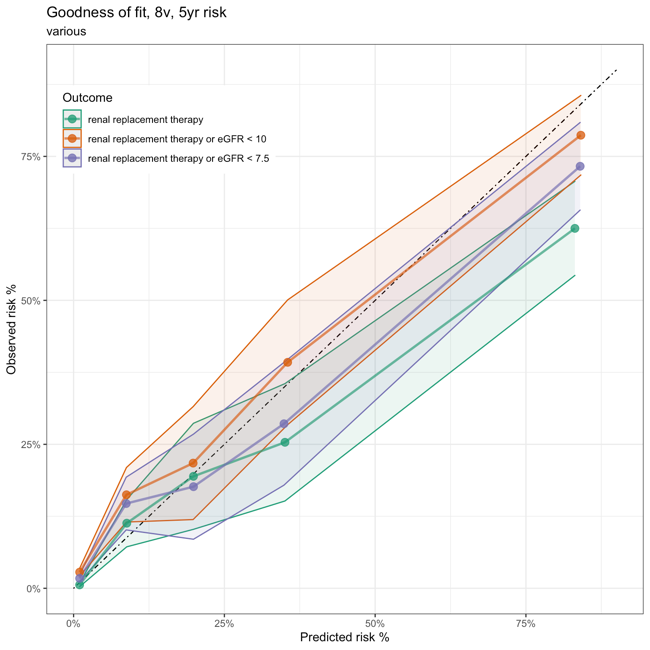
*

*Figure s4: Sensitivity analysis for goodness of fit from different outcomes. This demonstrates the eGFR < 7.5* mL/min/1.73m^2^ has the best model fit with the line being closest to the 45 degree angle.

**
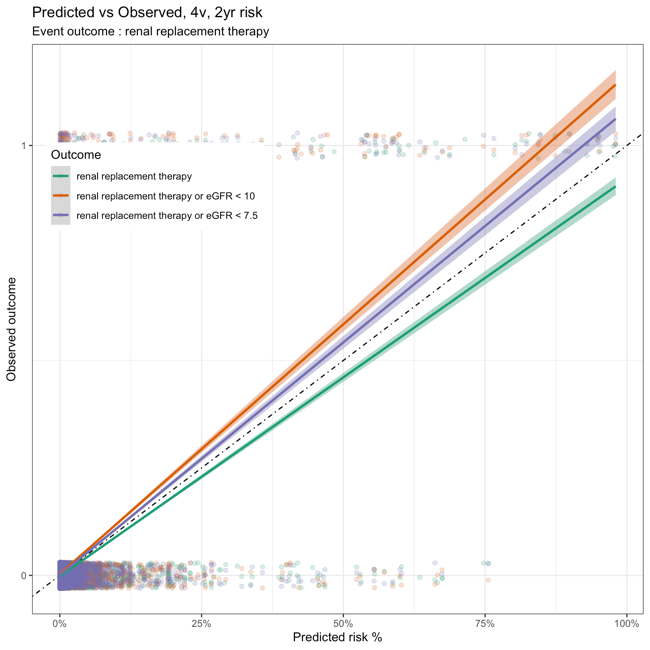
**
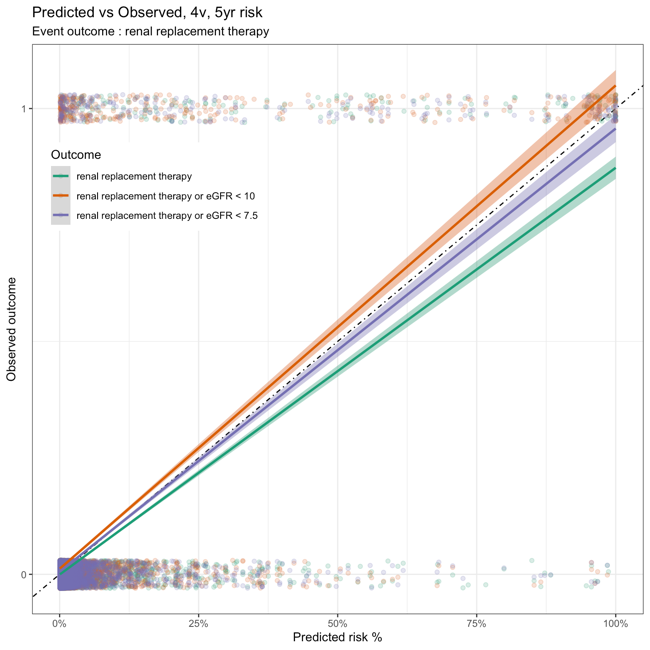


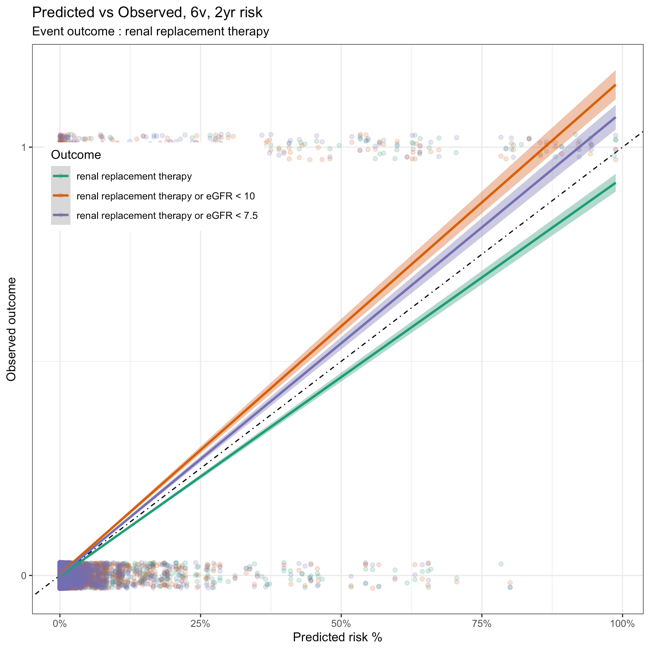

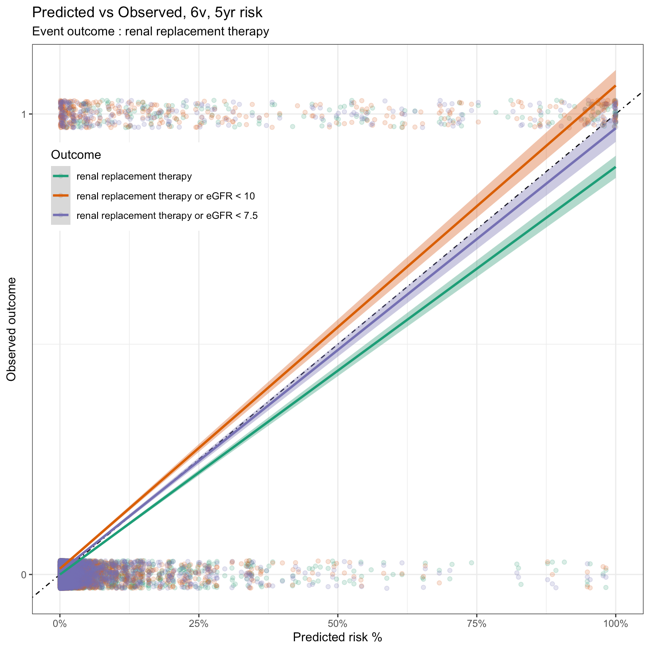


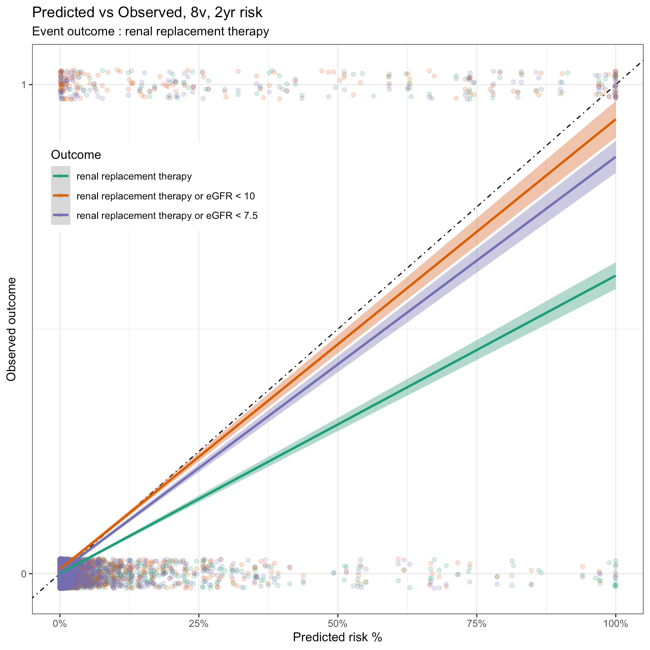

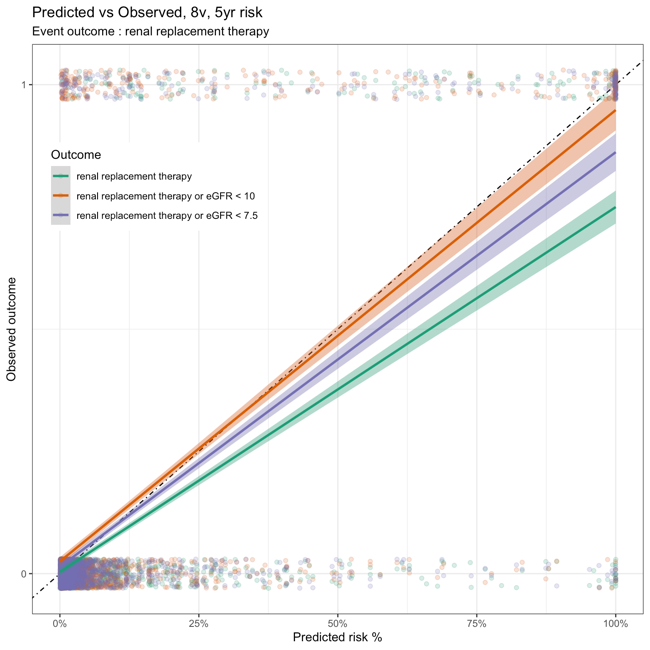


*Figure s5: Sensitivity Analysis demonstrates calibration for the different KFRE models at 2 and 5 years. The 4 and 6 variable risk score were better calibrated than the 8 variable risk score.*


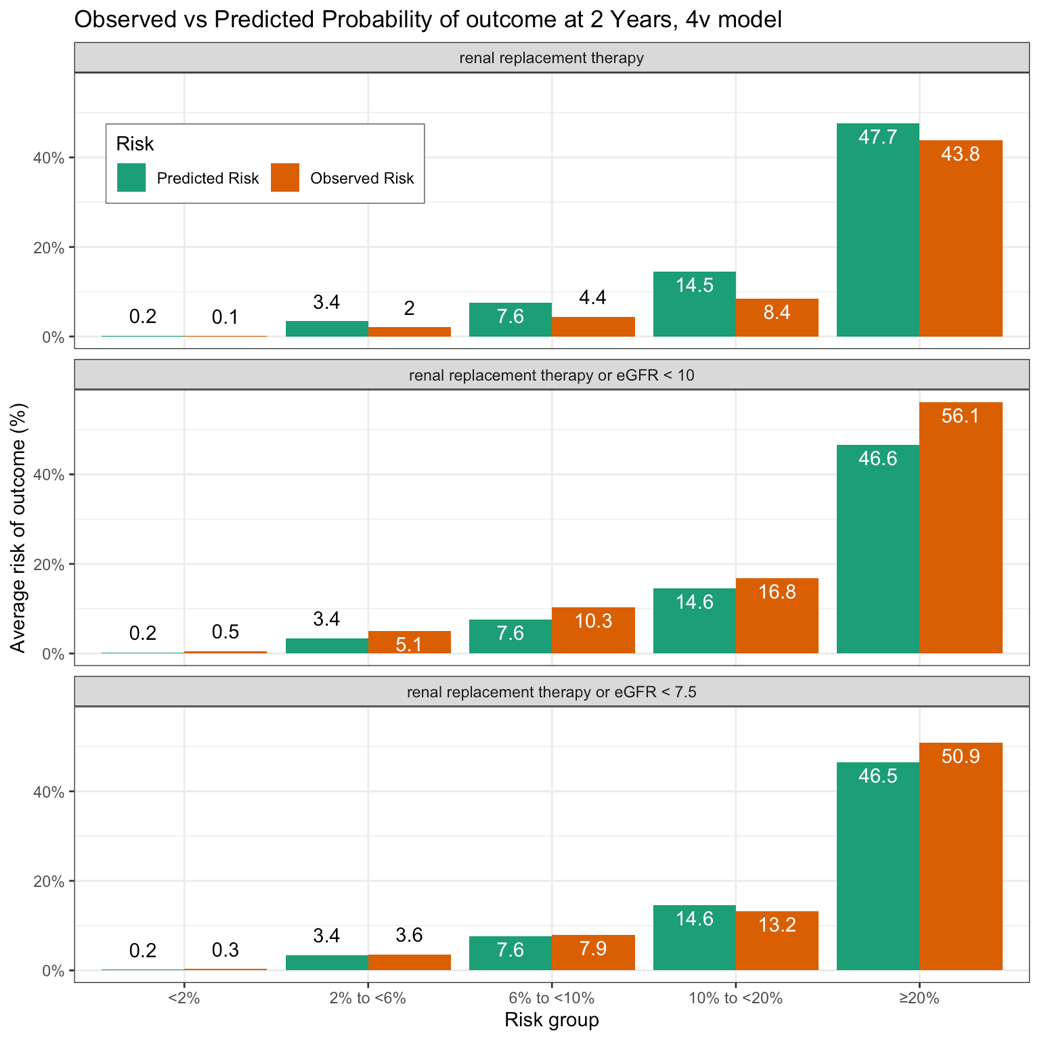


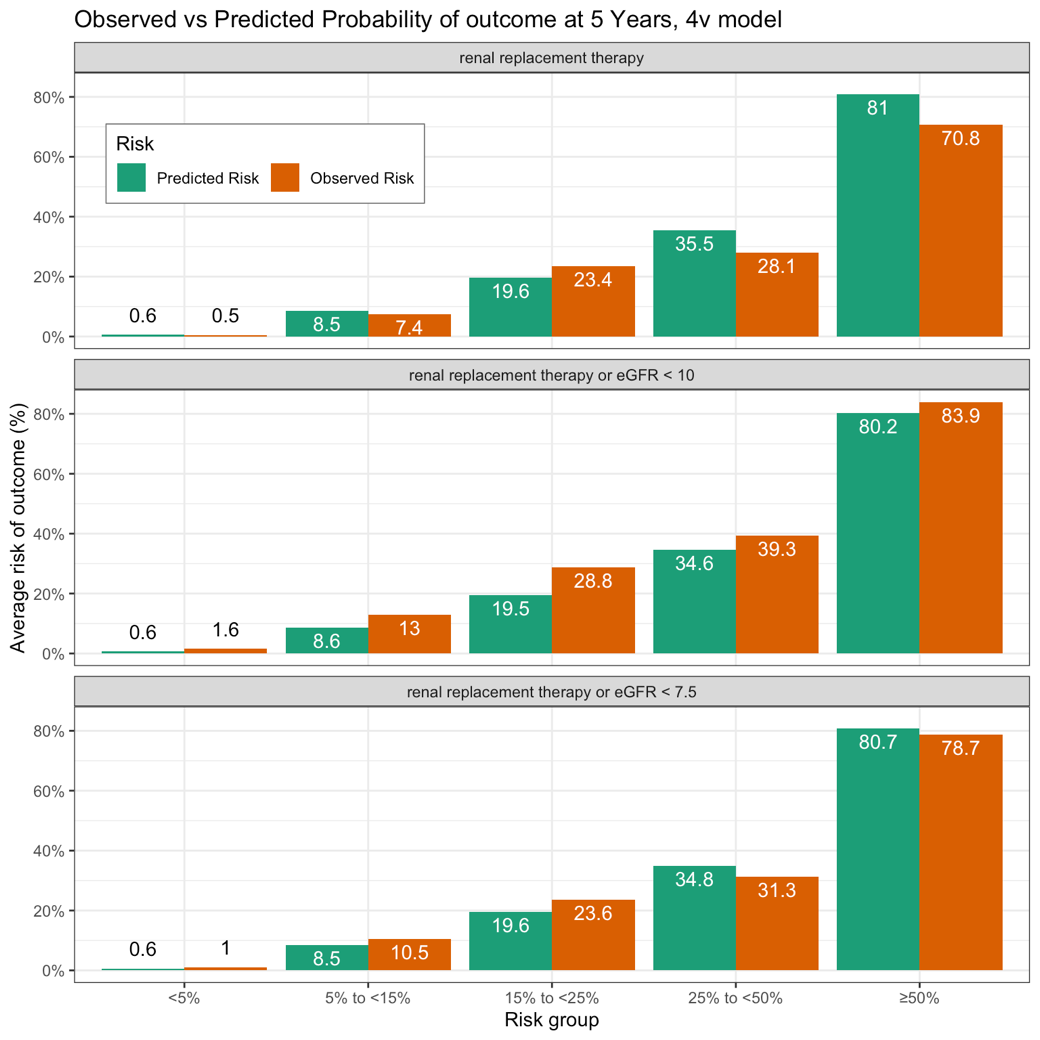


*Figure s6: Sensitivity Analysis for different outcomes demonstrates calibration for 4 variable KFRE models at 2 and 5 years by risk group.*

**
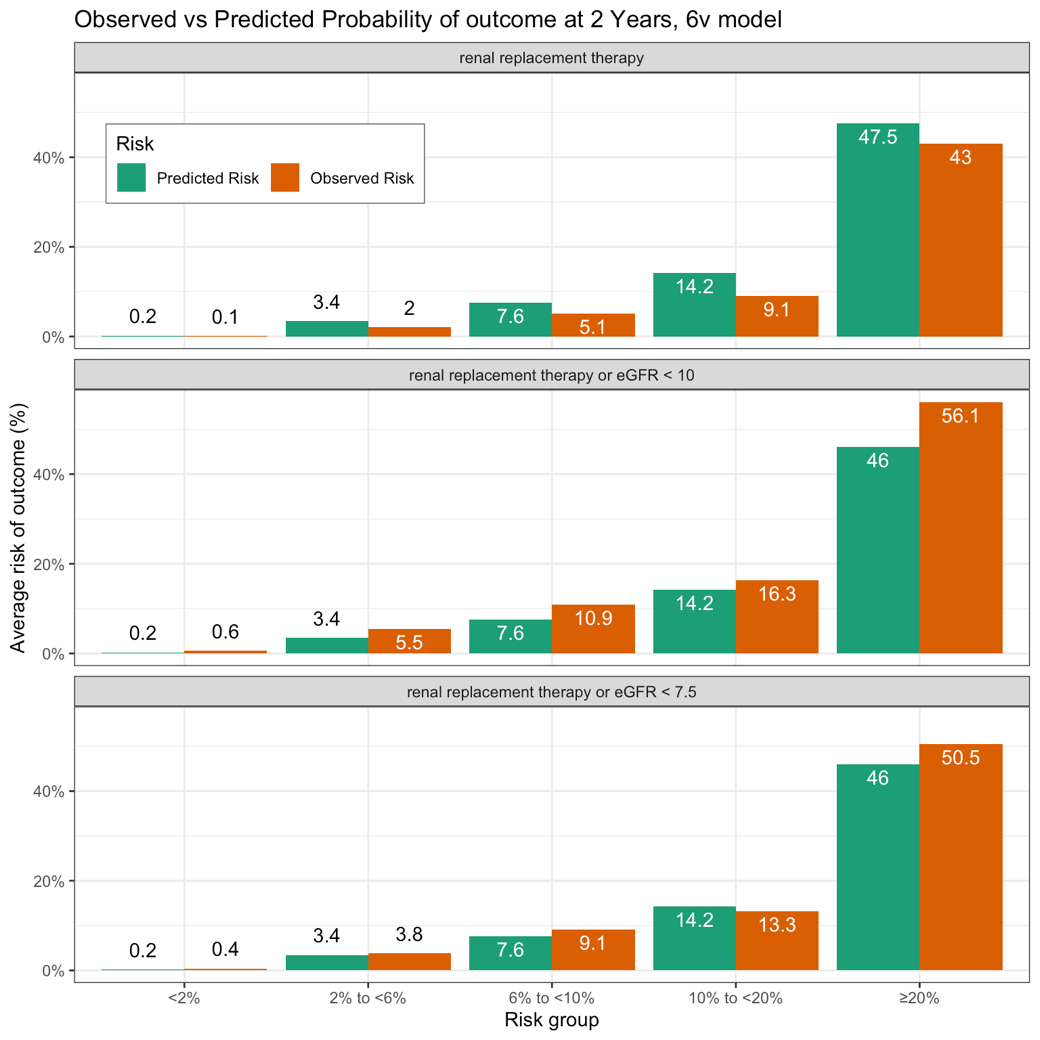
**

**
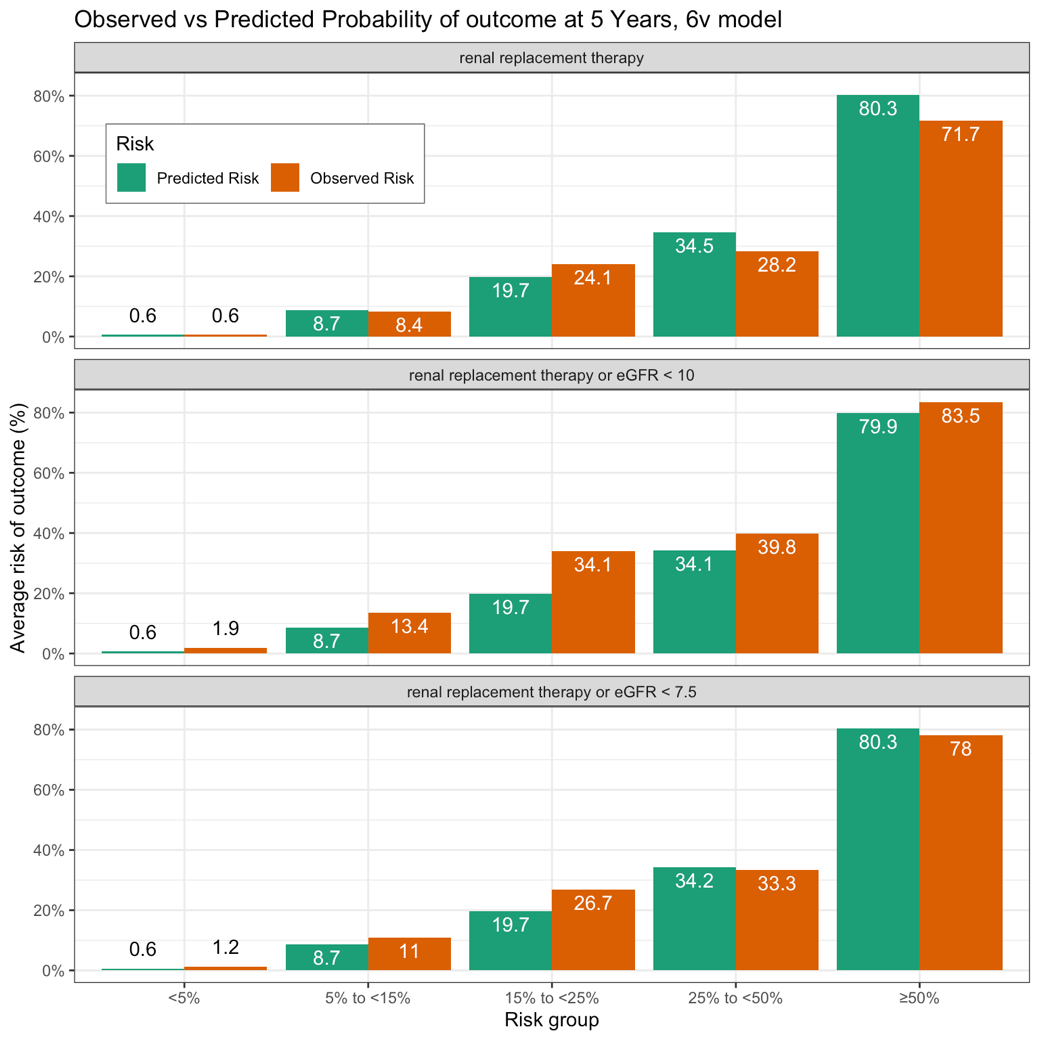
**

*Figure s7: Sensitivity Analysis demonstrates calibration for 6 variable KFRE models at 2 and 5 years by risk group.*

**
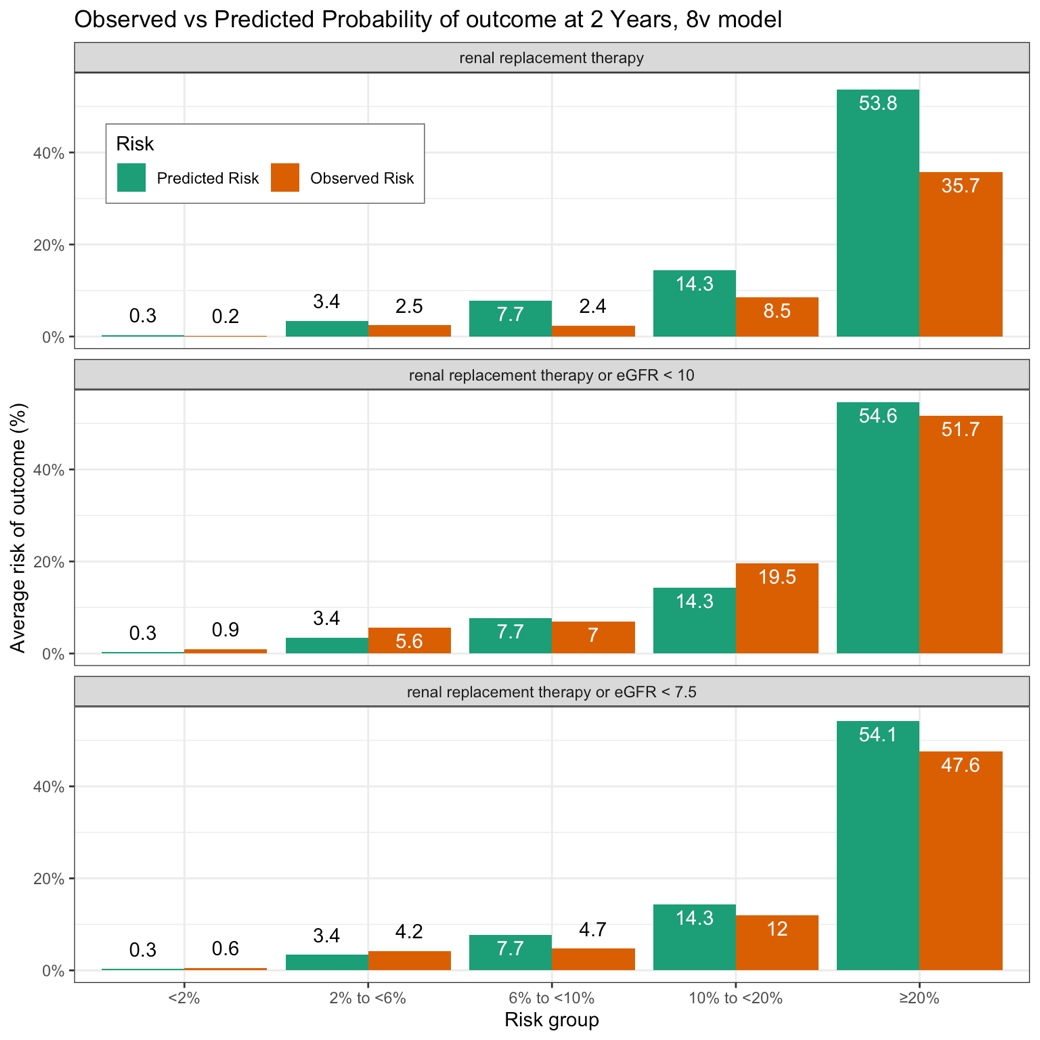
**

*
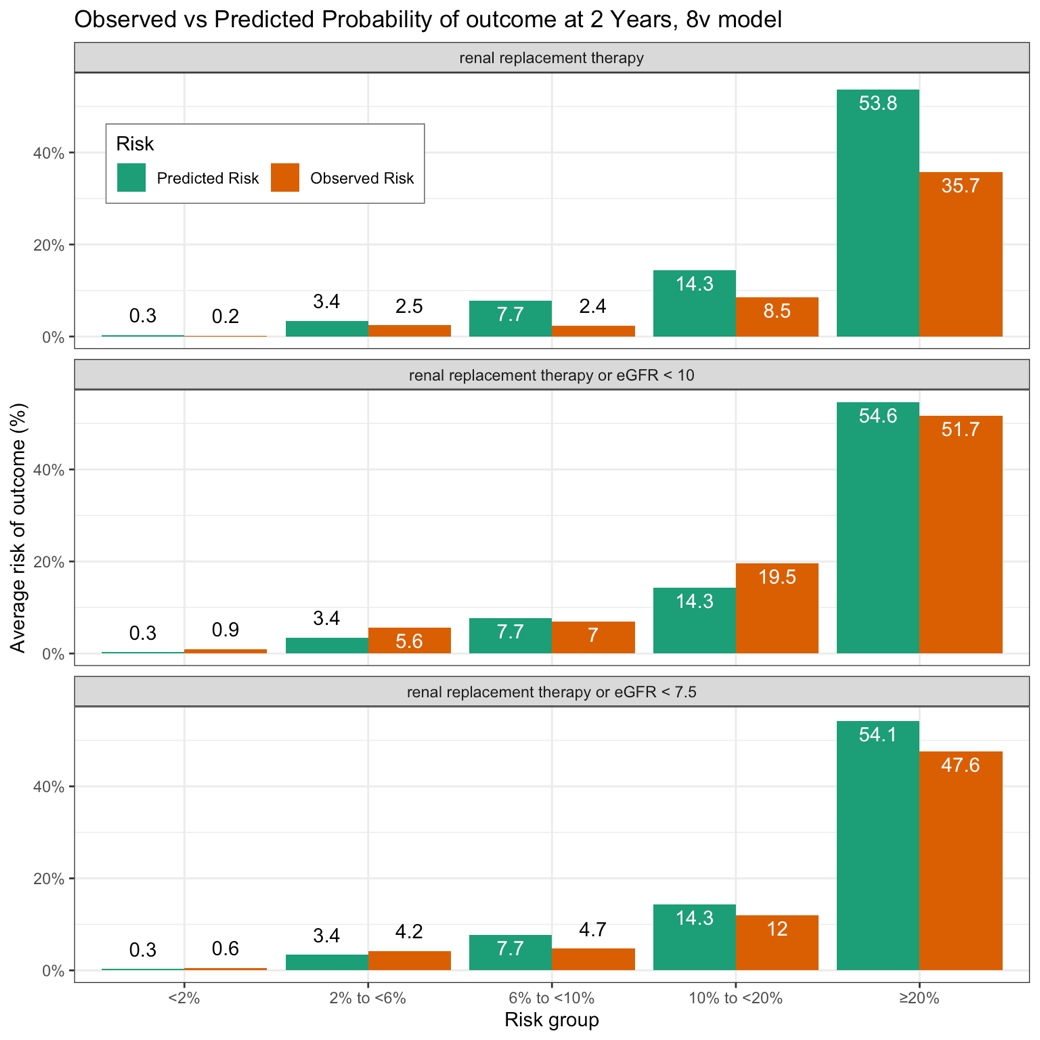
*

*Figure s8: Sensitivity Analysis demonstrates calibration for 8 variable KFRE models at 2 and 5 years by risk group.*

**
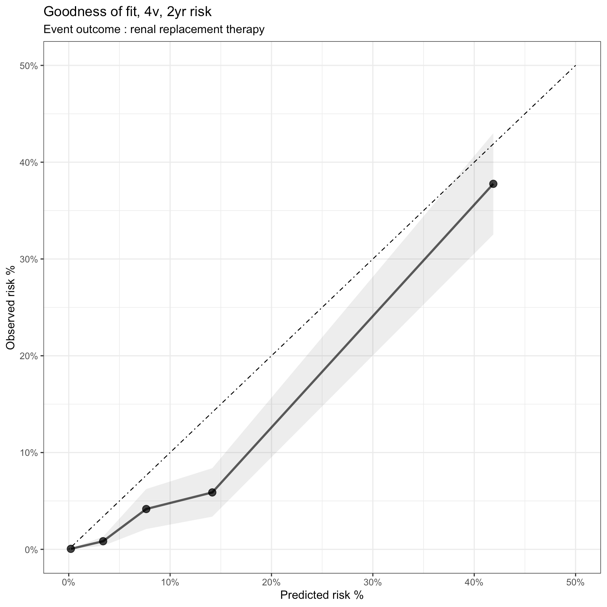

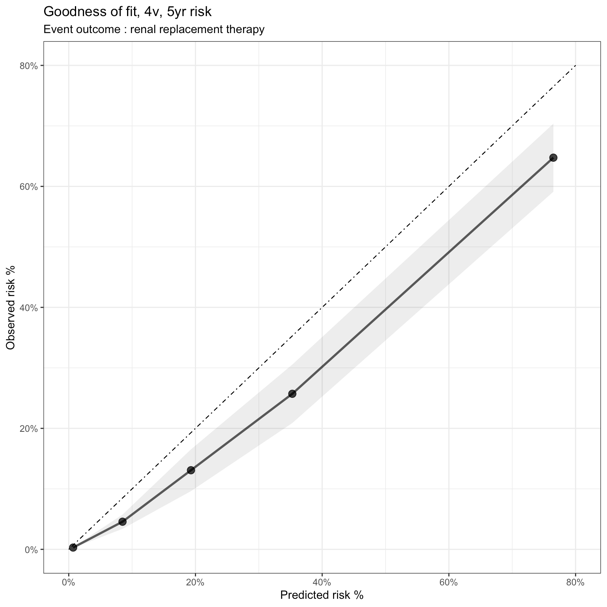
**

**
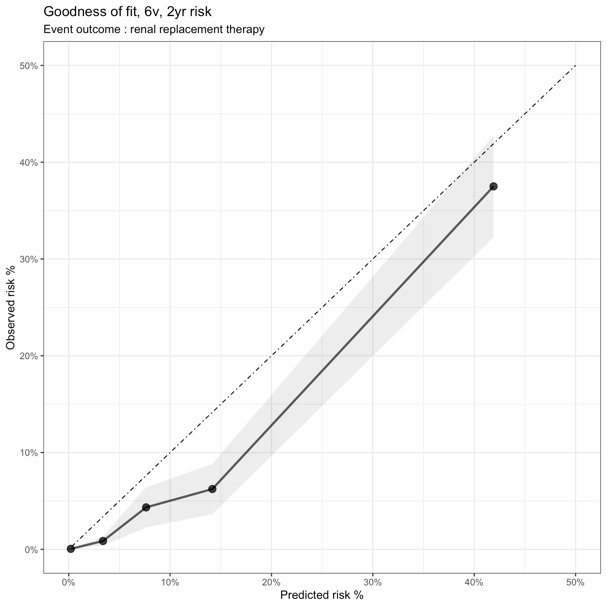

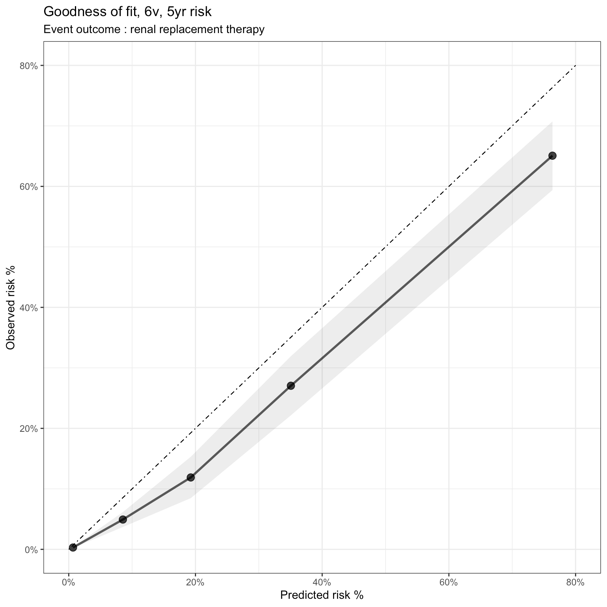
**

**
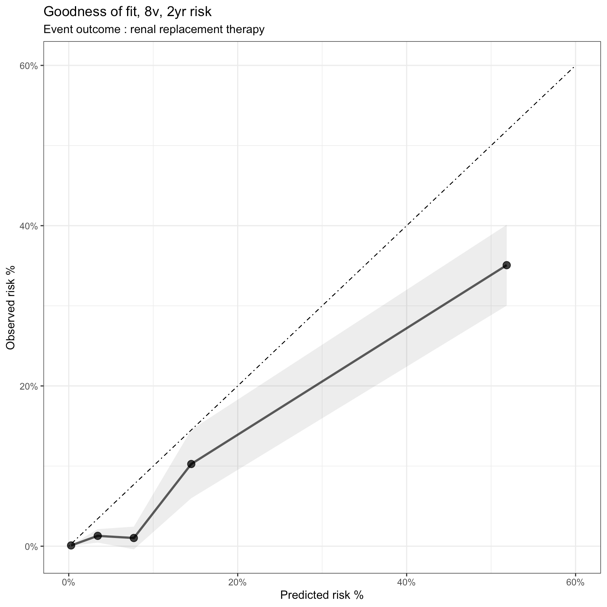

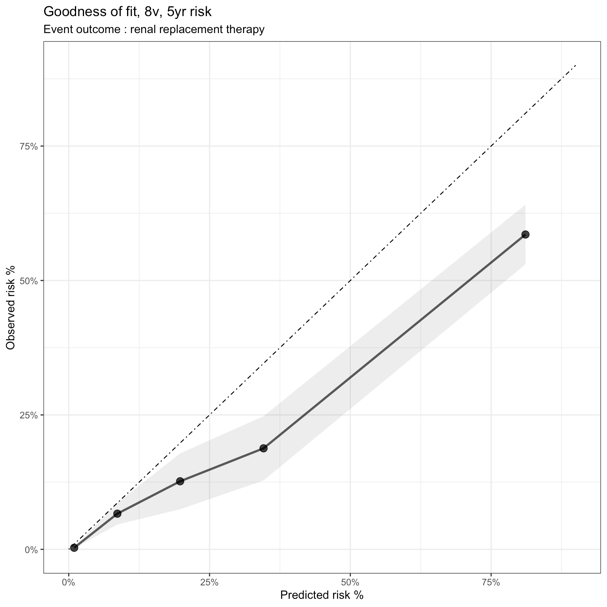
**

*Figure s9:Goodness of fit for the 4,6 and 8-variable KFRE at 2 and 5 years with multiple imputation .*

*
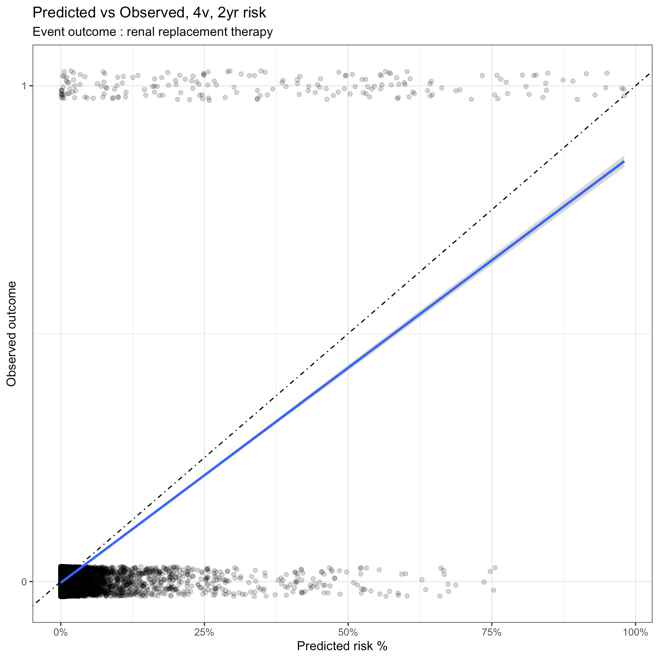

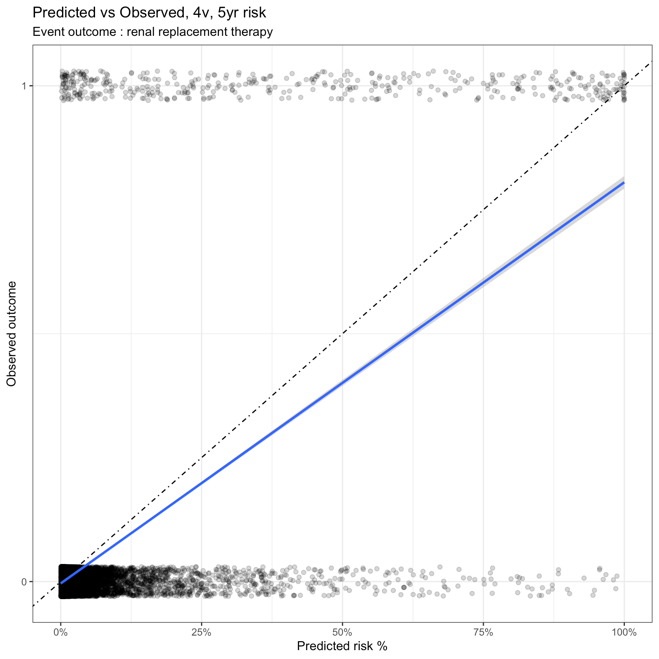
*


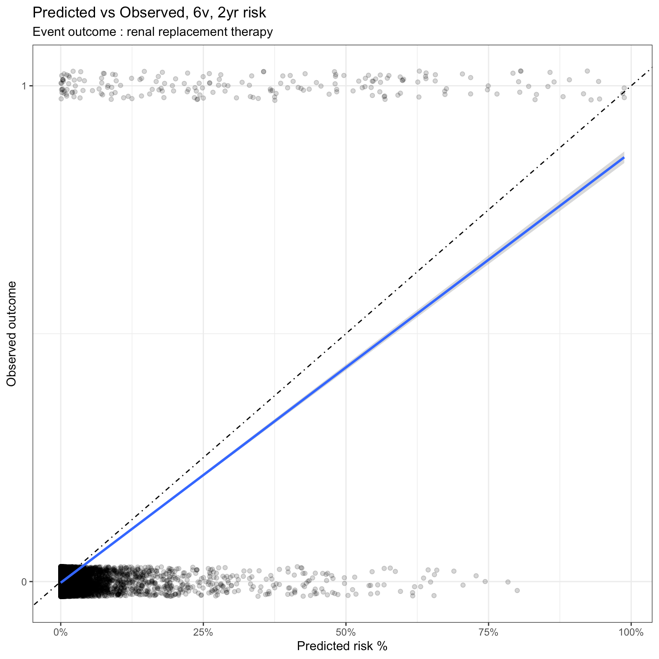

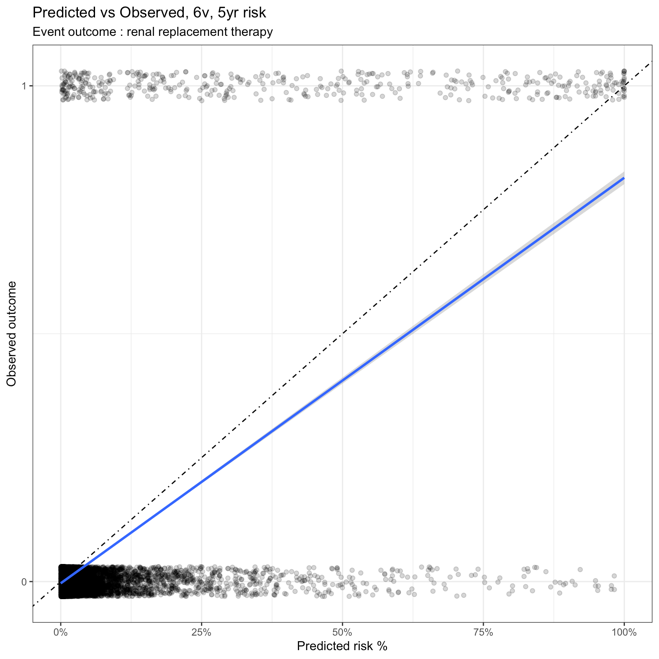


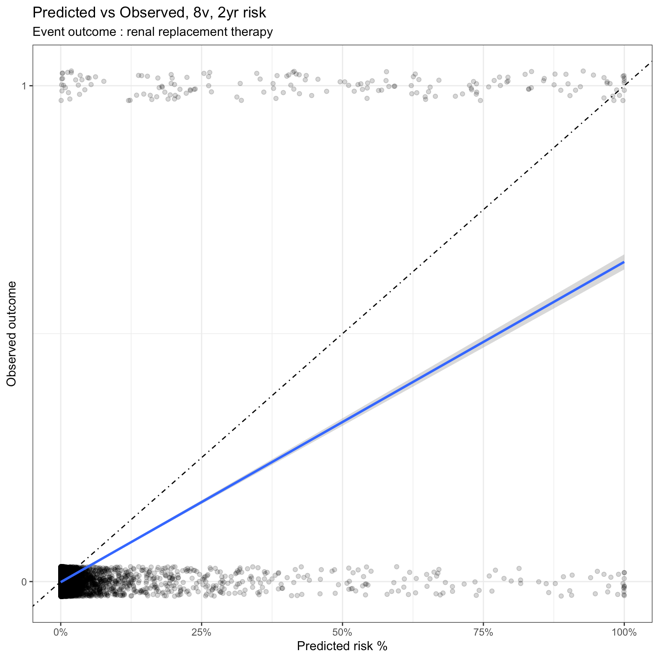

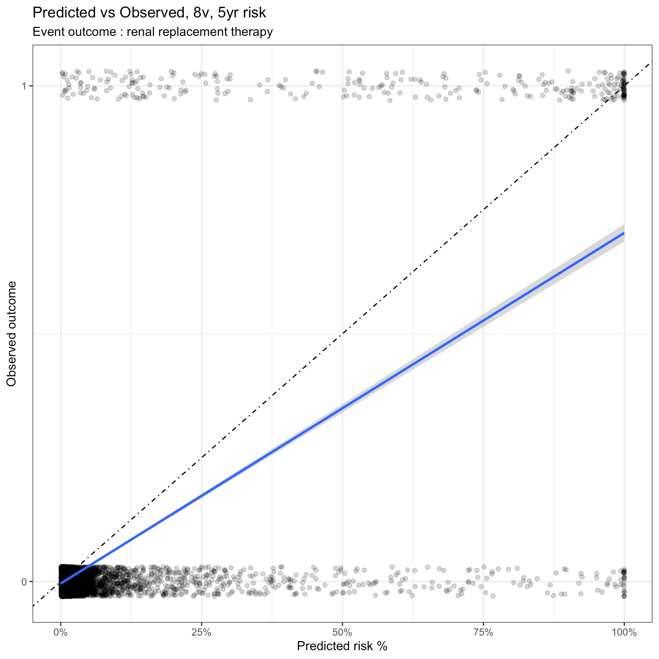


*Figure s10: Calibration for the 4,6, and 8-variable KFRE at 2 and 5 years with multiple imputation. The predicted risk is worse than the observed risk for all variables and time points, however is most miscalibrated for the 8-variable KFRE.*


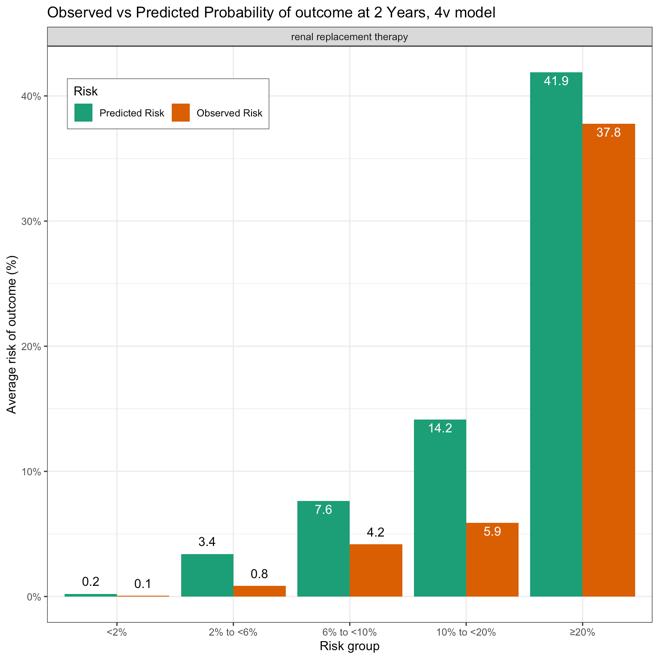

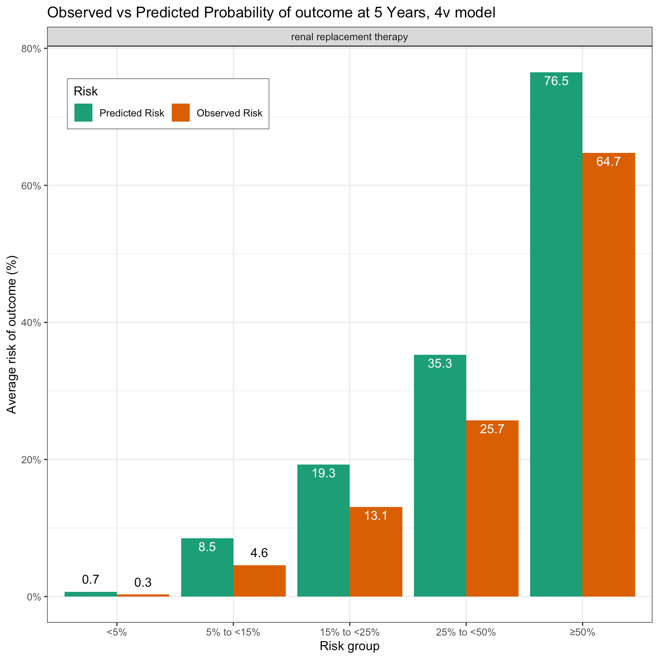


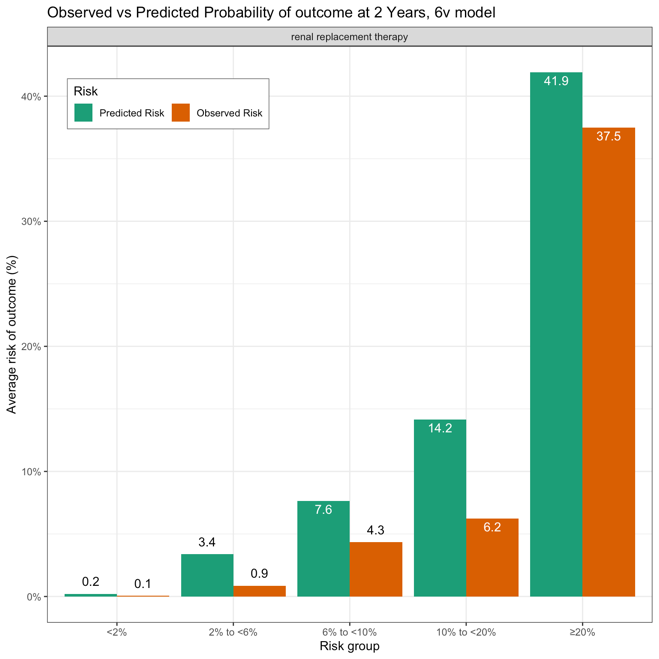

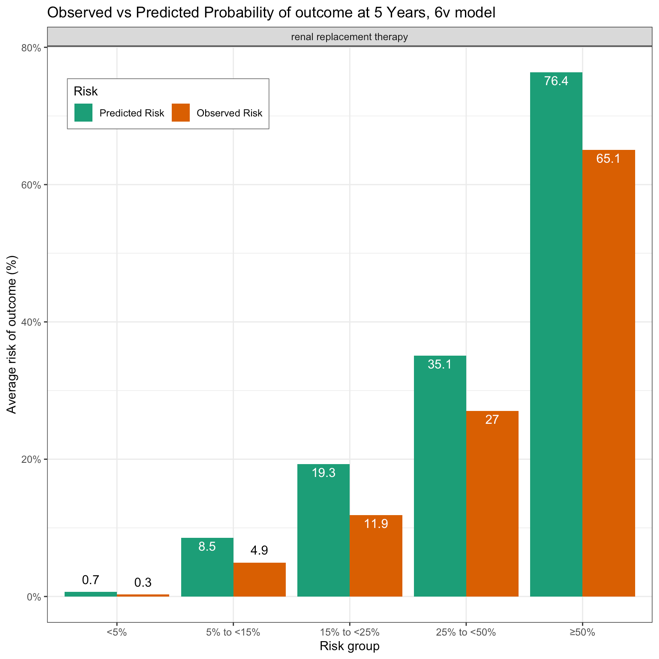


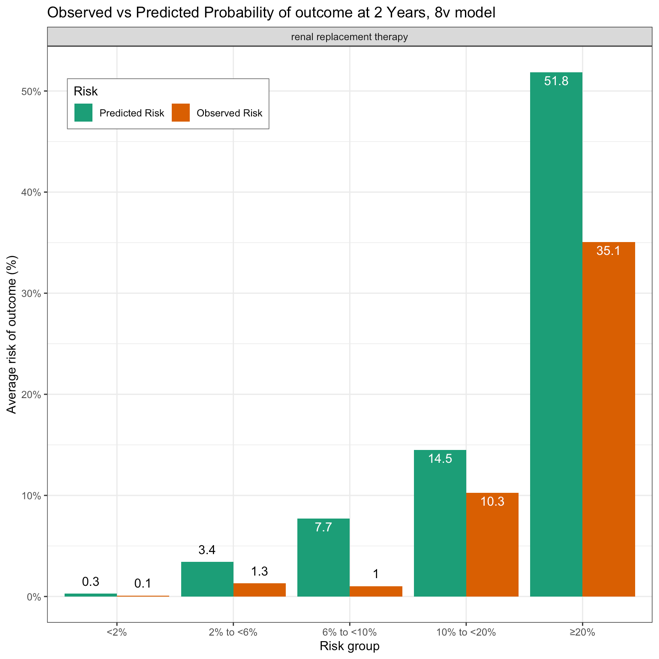

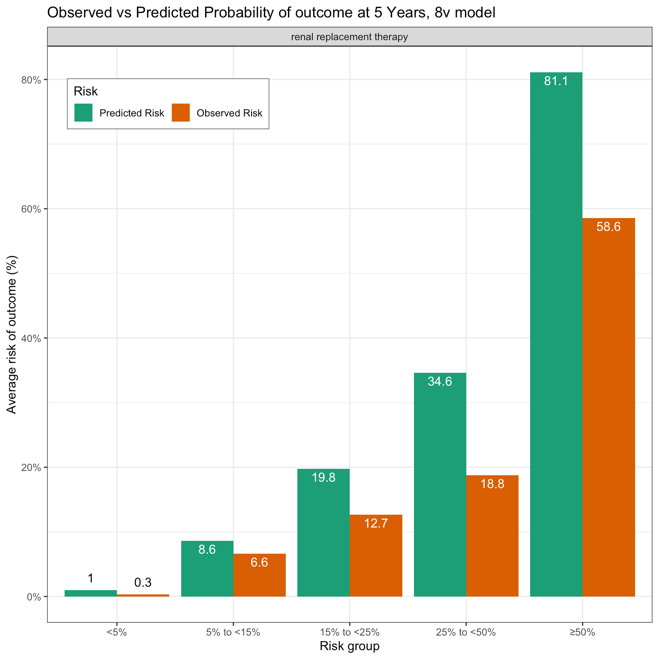


*Figure s11: Calibration by risk group for the 4,6, and 8-variable KFRE at 2 and 5 years with multiple imputation . The predicted risk is worse than the observed risk for all variables and time points, however is most miscalibrated for the 8-variable KFRE.*
